# Sexual and reproductive health among forcibly displaced persons in urban environments in low and middle-income countries: scoping review findings

**DOI:** 10.1186/s12978-024-01780-7

**Published:** 2024-04-12

**Authors:** Carmen H. Logie, Frannie MacKenzie, Kalonde Malama, Nicole Lorimer, Anoushka Lad, Michelle Zhao, Manjulaa Narasimhan, Sasha Fahme, Bülent Turan, Julia Kagunda, Kelika Konda, Aryssa Hasham, Amaya Perez-Brumer

**Affiliations:** 1 Factor-Inwentash Faculty of Social Work, University of Toronto, 246 Bloor Street W, Toronto, M5S 1V4 Canada; 2 Centre for Gender and Sexual Health Equity, Vancouver, Canada; 3 Women’s College Research Institute, Women’s College Hospital, Toronto, Canada; 4 United Nations University Institute for Water, Environment, and Health, Hamilton, Canada; 5 Department of Sexual and Reproductive Health and Research, including the UNDP/UNFPA/UNICEF/WHO/World Bank Special Programme of Research, Development and Research Training in Human Reproduction (HRP), World Health Organization, Geneva, Switzerland; 6 Center for Global Health, Weill Cornell Medicine, New York, NY USA; 7 Department of Health Promotion and Community Health, Faculty of Health Sciences, American University of Beirut, Beirut, Lebanon; 8 Department of Psychology, Koc University, Istanbul, Turkey; 9 Elim Trust, Nairobi, Kenya; 10 Daystar University, Nairobi, Kenya; 11 Universidad Peruana Cayetano Heredia, Lima, Peru; 12 Department of Population and Public Health Sciences, Keck School of Medicine of USC, University of Southern California, Los Angeles, USA; 13 Dalla Lana School of Public Health, University of Toronto, Toronto, Canada

**Keywords:** Refugee, Forcibly displaced, Sexual health, Reproductive health, Low and middle income country, Urban, Cities

## Abstract

**Background:**

Most forcibly displaced persons are hosted in low- and middle-income countries (LMIC). There is a growing urbanization of forcibly displaced persons, whereby most refugees and nearly half of internally displaced persons live in urban areas. This scoping review assesses the sexual and reproductive health (SRH) needs, outcomes, and priorities among forcibly displaced persons living in urban LMIC.

**Methods:**

Following The Joanna Briggs Institute scoping review methodology we searched eight databases for literature published between 1998 and 2023 on SRH needs among urban refugees in LMIC. SHR was operationalized as any dimension of sexual health (comprehensive sexuality education [CSE]; sexual and gender based violence [GBV]; HIV and STI prevention and control; sexual function and psychosexual counseling) and/or reproductive health (antental, intrapartum, and postnatal care; contraception; fertility care; safe abortion care). Searches included peer-reviewed and grey literature studies across quantitative, qualitative, or mixed-methods designs.

**Findings:**

The review included 92 studies spanning 100 countries: 55 peer-reviewed publications and 37 grey literature reports. Most peer-reviewed articles (*n* = 38) discussed sexual health domains including: GBV (*n* = 23); HIV/STI (*n* = 19); and CSE (*n* = 12). Over one-third (*n* = 20) discussed reproductive health, including: antenatal, intrapartum and postnatal care (*n* = 13); contraception (*n* = 13); fertility (*n* = 1); and safe abortion (*n* = 1). Eight included both reproductive and sexual health. Most grey literature (*n* = 29) examined GBV vulnerabilities. Themes across studies revealed social-ecological barriers to realizing optimal SRH and accessing SRH services, including factors spanning structural (e.g., livelihood loss), health institution (e.g., lack of health insurance), community (e.g., reduced social support), interpersonal (e.g., gender inequitable relationships), and intrapersonal (e.g., low literacy) levels.

**Conclusions:**

This review identified displacement processes, resource insecurities, and multiple forms of stigma as factors contributing to poor SRH outcomes, as well as producing SRH access barriers for forcibly displaced individuals in urban LMIC. Findings have implications for mobilizing innovative approaches such as self-care strategies for SRH (e.g., HIV self-testing) to address these gaps. Regions such as Africa, Latin America, and the Caribbean are underrepresented in research in this review. Our findings can guide SRH providers, policymakers, and researchers to develop programming to address the diverse SRH needs of urban forcibly displaced persons in LMIC.

**Plain English summary:**

Most forcibly displaced individuals live in low- and middle-income countries (LMICs), with a significant number residing in urban areas. This scoping review examines the sexual and reproductive health (SRH) outcomes of forcibly displaced individuals in urban LMICs. We searched eight databases for relevant literature published between 1998 and 2023. Inclusion criteria encompassed peer-reviewed articles and grey literature. SRH was defined to include various dimensions of sexual health (comprehensive sexuality education; sexual and gender-based violence; HIV/ STI prevention; sexual function, and psychosexual counseling) and reproductive health (antenatal, intrapartum, and postnatal care; contraception; fertility care; and safe abortion care). We included 90 documents (53 peer-reviewed articles, 37 grey literature reports) spanning 100 countries. Most peer-reviewed articles addressed sexual health and approximately one-third centered reproductive health. The grey literature primarily explored sexual and gender-based violence vulnerabilities. Identified SRH barriers encompassed challenges across structural (livelihood loss), health institution (lack of insurance), community (reduced social support), interpersonal (gender inequities), and individual (low literacy) levels. Findings underscore gaps in addressing SRH needs among urban refugees in LMICs specifically regarding sexual function, fertility care, and safe abortion, as well as regional knowledge gaps regarding urban refugees in Africa, Latin America, and the Caribbean. Self-care strategies for SRH (e.g., HIV self-testing, long-acting self-injectable contraception, abortion self-management) hold significant promise to address SRH barriers experienced by urban refugees and warrant further exploration with this population. Urgent research efforts are necessary to bridge these knowledge gaps and develop tailored interventions aimed at supporting urban refugees in LMICs.

**Supplementary Information:**

The online version contains supplementary material available at 10.1186/s12978-024-01780-7.

## Background

As of mid-2022, the global number of forcibly displaced individuals reached an estimate of 103 million [1], a significant majority of this population (53.2 million individuals) are internally displaced [1]. While approximately one-third, totaling 32.5 million people, hold recognized refugee status, another 4.9 million individuals are actively seeking asylum in another country [1, 2]. Forcibly displaced persons may experience poorer sexual and reproductive (SRH) outcomes than non-displaced persons due to the interplay of complex social ecological factors [3]. For instance, forcibly displaced persons may be exposed to sexual and gender-based violence (GBV) before, during, and/or following displacement, and/or upon resettlement. Further, they may experience reduced access to SRH services, including contraception and sexually transmitted infection (STI) prevention and treatment, due to poverty, socio-cultural differences, language, and literacy barriers [4–7]. Social and structural barriers such as intersectional stigma related to forcibly displaced status, gender, age, and limited SRH literacy can further constrain SRH engagement [8, 9].

Low- and middle-income countries (LMIC) host 74% of the globally forcibly displaced population, and it is estimated that the majority of refugees and nearly half (48%) of internally displaced people live in urban areas [1, 2, 10]. There is the potential that forcibly displaced persons residing in urban settings LMIC may live in poorer housing conditions with less economic and social support than those living in refugee camps or refugee settlement environments managed by humanitarian agencies [11, 12]. For instance, challenges facing forcibly displaced persons living in urban LMIC contexts can include transportation costs, higher living costs that may result in overcrowded living conditions, poverty, and language barriers to accessing relevant employment, education, health and other services [13–15]. It is plausible that these factors can also reduce the accessibility and utilization of SRH services. Inadequate SRH service provision is associated with increased gender-based violence (GBV), elevated risks for acquisition and transmission of HIV and other STIs, unintended pregnancies, and unsafe abortions [8, 16]. Further, urbanization among refugees may contribute to poverty and exacerbate gender inequities, both associated with increased likelihood of GBV [3, 17, 18]. With rising urbanization among forcibly displaced persons, there is a need for greater understanding of the sexual and reproductive health (SRH) outcomes and priorities to inform tailored intervention and programs.

Existing systematic reviews have reported evidence-based approaches to improve antenatal, postnatal, and newborn health, HIV prevention and treatment outcomes, and uptake of family planning resources and services, for forcibly displaced persons at large [19, 20]. There is evidence that interpersonal, health-system, and socio-cultural factors shape access to SRH care among forcibly displaced peoples [21]. Literature has also documented relationships between climate migration and GBV, decreased maternal and neonatal health, and increased barriers to accessing and using SRH services [22]. While these important reviews document factors that shape SRH among forcibly displaced peoples at large, there remains a notable lack of research focused on forcibly displaced persons regarding SRH issues including GBV prevention, STI transmission and treatment, menstruation hygiene management, and disrupted access to SRH care [19, 22]. Further, findings have not distinguished between urban or refugee camp/settlement contexts, resulting in a lack of clarity regarding specific needs, priorites, and SRH outcomes among forcibly displaced persons in urban LMIC contexts.

The objective of this scoping review is to identify, critically appraise, and synthesize the literature on sexual and reproductive health needs, outcomes, and priorities of forcibly displaced persons living in urban LMICs. A comprehensive understanding of these dimensions and existing research gaps can inform future practice, research, and policy.

## Methods

The Joanna Briggs Institute methodology for scoping reviews was followed throughout this review [23]. A complete and comprehensive explanation of the methods used can be found in the published study protocol [24].

### Search strategy

Completed in January 2023, we searched eight databases, MEDLINE, EMBASE, PsycINFO, CINAHL, IBSS, ASSIA, SSCI, and Global Medicus Index, for literature published between 1998 and 2022 on SRH needs among forcibly displaced persons in LMIC. The search structure first grouped terms for each of urban, refugees, sexual health, low and middle-income countries, and reproductive health using the Boolean operator OR. Following this, terms for urban and refugees were combined using the Boolean operator AND, and terms for sexual health and reproductive health were combined using the Boolean operator OR. Lastly, the search terms for urban refugees, sexual health or reproductive health, and low and middle-income countries were combined using the Boolean operator AND – ((urban OR cities OR municipal) AND (refugee* OR displace* OR asylum)) AND ((sexual health OR gender-based violence OR sexually transmitted disease*) OR (reproductive health OR prenatal OR contraception)) AND (low income countries OR middle income countries OR developing nations). A detailed search strategy for all databases can be found in the Supplementary File 1. A grey literature search was also conducted in accordance with a search guide developed by Godin et al. [25].

### Study selection

The *study population* was a) any forcibly displaced person, following UNHCR’s definition that includes refugee, migrant, asylum seeker, or internally displaced persons forced to flee due to persecution, conflict, human rights violations, or other serious events disrupting order [1, 2], b) living in a LMIC as defined by the World Bank Atlas Method [26] and c) living in an urban context, including urban, semi-urban, city, metropolis, or if study location is listed as urban in the UN World Urbanization Prospects database of country-specific definitions of ‘urban’ [27]. SRH was operationalized as any dimension of sexual health (comprehensive sexuality education [CSE]; sexual and gender based violence [GBV]; HIV and STI prevention and control; sexual function and psychosexual counseling) and/or reproductive health (antental, intrapartum, and postnatal care; contraception counselling and provision; fertility care; safe abortion care) [28, 29].

We included peer-reviewed or gray literature studies using quantitative, qualitative, or mixed-methods designs focused on any dimension of sexual/reproductive health written in the English language. Studies were excluded if they a) did not include forcibly displaced persons; b) included migrants by choice; c) did not focus on SRH; d) were not based in urban contexts; e) had metadata not in English; and f) there was no full-text article available. Key subject terms were searched among websites of governmental, non-governmental, and international organizations working with forcibly displaced persons.

### Data extraction and analysis

Once both the database search and grey literature search were completed, data from included records were extracted by a reviewer into a spreadsheet. All records were uploaded on to Covidence systematic review software (VeritasHealth Innovation, Melbourne, Australia) and duplicates were removed. On Covidence, each record’s title and abstract were screened by at least 2 study team members for inclusion eligibility. The full texts of all included articles were further screened by two study team members. At this point, the reference list of each included article was manually hand searched. If a relevant article was found via hand search, it was entered into Covidence and put through the screening process as outlined. All discrepancies were reviewed by a third team member and/or discussed with all reviewers. Extracted data points included the record’s general characteristics, population, concept, context, main outcome measure, and key findings relevant to this review. Every record’s data extraction was examined by a second team member for accuracy. All data were then summarized and collated into the accompanying narrative summaries.

## Results

**Fig. 1:**
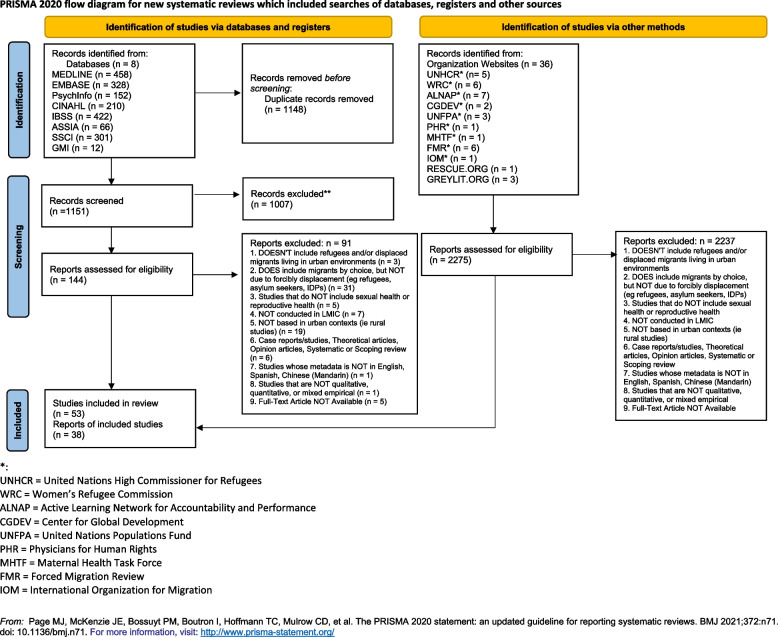
PRISMA flow diagram of a scoping review on urban forcibly displaced persons' sexual and reproductive health in low and middle-income countries

Our peer-reviewed article search returned 1151 results across eight databases and 2275 grey literature reports. In total, 92 documents including 55 peer-reviewed articles and 37 grey literature pieces met the inclusion criteria for this scoping review. Among the peer-reviewed articles, PRISMA Flow Chart in Fig. 1 shows the selection process for 53 peer-reveiewed articles (Fig. 1). Six additional peer-reviewed articles were hand searched, 2 of which met the inclusion criteria and were included.

The peer-reviewed articles were mapped onto dimensions of sexual health and reproductive health [29] (Table 1). The majority of peer-reviewed articles (*n* = 40; 72.7%) discussed sexual health domains including: GBV prevention, support and care (*n* = 23); HIV and STI prevention and control (*n* = 21); and comprehensive sexuality education (*n* = 12). Under the sexual health domain, no articles were located that discussed sexual function and psychosexual counselling. More than one-third (*n* = 20; 36.3%) discussed reproductive health areas including: antenatal, intrapartum and postnatal care (*n* = 13); contraception counselling & provision (*n* = 13); fertility care (*n* = 1); and safe abortion care (*n* = 1). While not within the SRH framework [28, 29], menstrual hygiene management was included as a SRH issue in this review as it was discussed in three articles. Eight articles discussed intervention areas that included both reproductive and sexual health domains. Sexual and reproductive health dimensions covered in peer-reviewed articles are displayed in Table 2.

**Table 1:** Overview of peer-reviewed articles (*n* = 55) included in scoping review on sexual and reproductive health of urban forcibly displaced persons in low and middle-income countries

Author(s) / year	Region	Study Design	Sample / Population	SRH focus	Study Objectives
Abbasi-Kangevari et al. (2020) [30]	Tehran, Iran	Quantitative	*n* = 231, pregnant Syrian refugee women	Antenatal care	To assess the utilization of private and public antenatal care among Syrian refugees in Tehran
Agadjanian (1998) [31]	Maputo, Mozambique	Qualitative	*n* = 170, Internally displaced women	Reproductive behaviour	To examine the impact of internal displacement on reproductive and socio-economic behaviours
Al-Modallal (2016) [32]	Amman, Irbid, and Zarqa, Jordan	Quantitative	*n* = 238, Palestinian refugee women	SGBV/IPV	To identify the relationship between psychological, physical, and sexual partner violence and physical health problems in refugee women
Al-Modallal et al. (2015) [33]	Amman, Irbid, and Zarqa, Jordan	Quantitative	*n* = 300, Palestinian refugee women	SGBV/IPV	To examine the prevalence of IPV and identify demographic factors that increase risk or protection against IPV victimization among Palestinian women in Jordan
Bahamondes et al. (2022) [34]	Roraima, Brazil	Mixed Methods	*N* = 12,436 women who had given birth in Roraima, healthcare workers and policy makers	Antenatal care, postnatal care	To assess the availability and access to SRH services for Venezuelan migrants during COVID-19
Balsara et al. (2010)	Haripur, Pakistan	Quantitative	*n* = 634, Afghan refugee women	Reproductive tract disorders	To identify commonly occurring reproductive tract infections (RTIs), describe knowledge of women about RTIs, and assess physical and behavioural factors contributing to the development of RTIs
Benage et al. (2015) [35]	Lebanon	Quantitative	*N* = 420, pregnant Syrian refugee women	Antenatal care	To describe antenatal care access, the scope of existing antenatal care, and antenatal and family planning behaviors and practice among pregnant Syrian refugees in various living conditions and multiple geographic areas of Lebanon
Campbell et al. (2016) [36]	Port Au Prince, Haiti	Qualitative	*N* = 208, internally displaced Haitian women	SGBV/IPV	To determine the effect of GBV and its impacts on health among young women in Port Au Prince before and after the 2010 Haitian earthquake
Cardoso et al. (2016) [37]	Abidjan, Côte d’Ivoire	Qualitative	*n* = 91, internally displaced and non-internally displaced men and women	SGBV/IPV	To examine the social and structural characteristics of the urban environment that contributed to the IPV experiences of women residing in post-conflict Abidjan, Côte d’Ivoire
Dadras et al. (2020) [38]	Tehran, Iran	Qualitative	*N* = 30, pregnant Afghan refugee women	Antenatal care	To explore the potential barriers to prenatal care among Afghan women in Iran
Daw et al. (2022)	Libya	Quantitative	*N* = 4,539, internally displaced Libyan men and women	HIV	To determine the impact of conflict on HIV transmission and epidemiology among conflict affected Libyans
DeJong et al. (2017) [39]	Bar Elias and Qabelias, Lebanon	Qualitative	*N* = 118, Syrian refugee adolescents and their parents	SGBV/IPV	To understand the specific experiences of very young adolescents, those 10–14 years of age, in a protracted crisis context
Delkhosh et al. (2019) [40]	Semnan, Iran	Quantitative	*N* = 188, Afghan refugee women	SGBV/IPV	To determine the prevalence of IPV among Afghan refugee women in a settlement in Iran and its impact on reproductive health outcomes
Feseha et al. (2012) [41]	Tigray Regional State, Ethiopia	Quantitative	*N* = 422, Eritrean refugee women	SGBV/IPV	To assess the magnitude of intimate partner physical violence and associated factors among women in Shimelba refugee camp, Northern Ethiopia
Hammoury et al. (2009) [42]	Sidon, Lebanon	Quantitative	*N* = 351, pregnant Palestinian refugee women	SGBV/IPV	To determine the factors associated with domestic violence against pregnant Palestinian refugee women residing in Lebanon and currently using the United Nation Relief and Work Agency's (UNRWA) primary healthcare services
Kabakian-Khasholian et al. (2017) [43]	Bekaa, Lebanon	Qualitative	*N* = unspecified, Syrian refugee women and healthcare providers	Family planning	To explore the perspectives of women and service providers about fertility behaviour of and service provision to Syrian refugee women
Khatoon et al. (2018) [44]	Nepal	Quantitative	*N* = 323, Bhutanese Refugees	HIV	To describe the socio-demographic characteristics, assess the utilization of HIV testing and counselling services, and to explore the reasons for the non-utilization of HIV testing and counselling services among the key populations at the Bhutanese refugee camps
Khawaja & Barazi (2005) [45]	Jordan	Quantitative	*N* = 395, refugee men and women	SGBV/IPV	To examine the similarity between men’s self-reports of violence and women’s reports of being subjected to domestic violence
Khawaja & Hammoury (2008) [46]	Sidon, Lebanon	Quantitative	*N* = 349, pregnant refugee women	SGBV/IPV	To examine the correlates of forced sexual intercourse among pregnant refugee women attending an antenatal clinic
Korri et al. (2021) [18]	Bourj Hammoud, Lebanon	Qualitative	*N* = 40, adolescent Syrian refugee girls	SRH literacy	To understand sexual and reproductive health perceptions and experiences among adolescent refugee girls in an urban setting
Krause et al. (2015)	Zaatri Camp and Irbid City, Jordan	Qualitative	*N* = 170, Syrian refugee women and key informants	Antenatal care, family planning, HIV, postnatal care, SGBV/IPV, SRH literacy, STIs	To determine the status of Minimum Initial Services Package (MISP) implementation for Syrian refugees in Jordan as part of a global evaluation of reproductive health in crises
Logie et al. (2019) (a) [47]	Kampala, Uganda	Quantitative	*N* = 333, displaced adolescent girls	SGBV/IPV	To examine the prevalence of young adulthood violence (YAV) and recent IPV among refugee and displaced adolescent girls, and the social factors associated at an intrapersonal, interpersonal, and community level
Logie et al. (2019) (b) [48]	Kampala, Uganda	Quantitative	*N* = 445, refugee and displaced adolescents	HIV	To assess the reliability of the SRH stigma scale while examining the gender differences in both HIV testing uptake and HIV service awareness and their association with stigma among urban and refugee youth living in Kampala's informal settlements
Logie et al. (2020) [49]	Kampala, Uganda	Quantitative	*N* = 445, refugee and displaced adolescents	STIs	To assess the prevalence of STI testing awareness, uptake, and diagnosis their impacting factors among urban refugee and displaced youth living in Kampala's informal settlements
Logie et al. (2021) (a) [13]	Kampala, Uganda	Quantitative	*N* = 412, refugee and displaced adolescents	HIV	To examine the relationships between HIV prevention and transactional sex among urban refugee and displaced youth in Kampala
Logie et al. (2021) (b) [50]	Kampala, Uganda	Qualitative	*N* = 44, refugee and displaced adolescents	HIV	To understand HIV testing experiences among urban refugee youth in Kampala, and their perspectives on HIV self-testing
Logie et al. (2021) (c) [51]	Kampala, Uganda	Qualitative	*N* = 44, refugee and displaced adolescents	HIV	To explore the experiences, preferences and engagement with HIV testing and prevention among urban refugee adolescents and youth in Kampala, Uganda, with a focus on the role of contextual factors in shaping access and uptake
Logie et al. (2022) (a) [24]	Kampala, Uganda	Qualitative	*N* = 44, refugee and displaced adolescents	HIV	To examine contextual factors that impact HIV testing and prevention based on the experiences of urban refugee and displaced adolescents and youth in Kampala
Logie et al. (2022) (b) [52]	Kampala, Uganda	Quantitative	*N* = 445, refugee adolescents	HIV, SGBV/IPV	To understand the interactions between frequent alcohol use, depression, and violence on HIV vulnerability among urban refugee youth
Logie et al. (2022) (c) [53]	Kampala, Uganda	Quantitative	*N* = 450, refugee adolescents	HIV	To examine the association of HIV testing and social contextual factors among urban refugee youth
López et al. (2010) [54]	Medellin, Colombia	Qualitative	*N* = 23, forcibly displaced women	HIV, STIs	To determine the social vulnerability to sexually transmitted diseases and AIDS in women forcibly displaced in Medellin
Malama et al. (2023) [55]	Kampala, Uganda	Quantitative	*N* = 333, refugee and displaced adolescent girls	Family planning	To determine the factors associated with motherhood among urban refugee adolescent girls and young women
Marquez-Lameda (2022) [56]	Peru	Quantitative	*N* = 3,378, Venezuelan migrant and refugee women	Family planning	To determine the influencing factors on Venezuelan migrant and refugee women's access to SRH services and contraceptive usage
Masterson et al. (2014) [57]	North Lebanon and the Bekaa Valley, Lebanon	Quantitative	*N* = 452, Syrian refugee women	Gynecologic health	To increase understanding of reproductive health concerns in a conflict setting by assessing the experiences of displaced women in Lebanon who have recently fled from the conflict in Syria
Mendelsohn et al. (2012) [58]	Kuala Lumpur, Malaysia	Quantitative	*N* = 301, refugee and asylum-seeking men and women, and host community members	HIV	HAART adherence and clinical outcomes among refugee and asylum seekers and local host community members in the same clinic in Kuala Lumpur
Mendelsohn et al. (2014) (a) [59]	Kuala Lumpur, Malaysia	Quantitative	*N* = 301, refugee and asylum-seeking men and women, and host community members	HIV	To compare HIV treatment outcomes in refugees and host community members accessing HAART in the same clinic in Kuala Lumpur
Mendelsohn et al. (2014) (b) [60]	Kakuma, Kenya and Kuala Lumpur, Malaysia	Qualitative	*N* = 26, refugee men and women	HIV	To document and examine accounts of the threats, barriers and facilitators experienced in relation to HIV treatment and care and to conduct comparisons across settings
Morof et al. (2014) [61]	Kampala, Uganda	Quantitative	*N* = 117, DR Congolese and Somalian refugee and asylum-seeking women	SGBV/IPV	Examine the prevalence of various types of violence among refugees and asylum seekers to determine the impact of GBV and mental illness on the public health system
Nabulsi et al. (2021)	Lebanon	Review	Syrian refugee women and girls	Family planning HIV, SGBV/IPV, STIs,	To explore the SRH response for Syrian refugee women and girls in Lebanon, with a focus on minimal initial service package (MISP) implementation
Okumu et al. (2022) [62]	Kampala, Uganda	Quantitative	*N* = 242, forcibly displaced adolescents	Family planning, sexting	To adapt a scale that examines psychometric properties of condom use experiences among forcibly displaced adolescents in Kampala, Uganda
Okumu et al. (2023) [63]	Kampala, Uganda	Quantitative	*N* = 242, forcibly displaced adolescents	Family planning	To identify the patterns of sexting among forcibly displaced adolescents in Kampala, Uganda
Olupot-Olupot et al. (2008) [64]	Teso, Northern Uganda	Qualitative	*N* = 51, conflict-affected HIV positive men and women, and healthcare workers	HIV	To determine patient and health worker concerns regarding antiretroviral adherence in a conflict-affected population
Pardhi et al. (2020) [65]	Mumbai, India	Qualitative	*N* = 15, pregnant migrant women with children under 2 y/o	Antenatal care	To examine the sanitation, hygiene and living conditions of migrants who were forced to leave their homes because of drought, focusing on the health problems of pregnant migrant women and their children
Patel et al. (2012) [17]	Gulu district, Uganda	Qualitative	*N* = 132, displaced adolescent girls and adult women	HIV, SRH literacy	To deepen the knowledge base on the distinct vulnerabilities of girls in time of conflict by qualitatively exploring the sexual vulnerabilities of adolescent girls surviving abduction and displacement in Northern Uganda
Patel et al. (2014) [66]	Gulu district, Uganda	Quantitative	*N* = 384, internally displaced adolescents and young adults	HIV	To assess the prevalence and correlates of HIV infection among young people living in post-conflict transition in Gulu District, northern Uganda
Rayamajhi et al. (2016) [67]	Eastern Nepal	Quantitative	*N* = 350, married Bhutanese refugee women	Family Planning	To find out the factors related to use of family planning methods by married women of reproductive age in the Bhutanese Refugee camps of eastern Nepal
Roupetz et al. (2020) [68]	Beirut, Beqaa and Tripoli, Lebanon	Qualitative	*N* = 112, Syrian refugee women, men, and girls, Lebanese and Palestinian men, and community leaders	SGBV/IPV	To analyze the threats and experiences of SGBV among Syrian refugee women and girls who are integrated into their host communities and to provide a nuanced perspective of SGBV that includes insights from male community members
Schmitt et al. (2017)	Rakhine State, Myanmar and Tripoli, Beirut and the Bekaa Valley, Lebanon	Qualitative	*N* = 265, displaced women and girls, and humanitarian staff	MHM	To explore the menstrual hygiene management barriers facing girls and women, and the various relevant sectoral responses being conducted
Sipsma et al. (2015) [69]	Rwanda	Quantitative	*N* = 548, ever married Congolese refugee women	SGBV/IPV	To examine patterns of conflict-related violence and intimate partner violence (IPV) and their associations with emotional distress among Congolese refugee women living in Rwanda
Tohme et al. (2016) (a) [70]	Beirut, Lebanon	Quantitative	*N* = 150, Palestinian, Iraqi, and Syrian refugee men who have sex with men (MSM)	HIV	To determine the prevalence and correlates of HIV testing and condom use among refugee MSM in Beirut
Tohme et al. (2016) (b) [71]	Beirut, Lebanon	Quantitative	*N* = 150, Palestinian, Iraqi, and Syrian refugee MSM	HIV	To examine the socio-demographic determinants of sexual risk behaviours and HIV testing among refugee MSM in Beirut
Yaman Sözbir et al. (2021) [72]	Turkey	Qualitative	*N* = 15, Syrian refugee women	Antenatal care, postnatal care	To describe the birth experiences of Syrian refugee women in Turkey
Wako et al. (2015) [73]	Rwanda	Quantitative	*N* = 548, ever married Congolese refugee women	SGBV/IPV	To describe the prevalence and correlates of past-year intimate partner violence (IPV) among displaced women
Wirtz et al. (2013) [74]	Addis Ababa, and Jijiga District, Ethiopia	Qualitative	*N* = 114, refugee women, and health protection and community service staff	SGBV/IPV	To identify the type, perpetrators, and location of gender-based violence among a population impacted by conflict
Wringe et al. (2019) [75]	Izmir, Turkey	Qualitative	*N* = 29, displaced adolescent and adult men and women	SGBV/IPV	To explore the risks of gender-based violence against Syrian adolescent girls and young women in Turkey and examine how these risks are shaped by their displacement

**Table 2:**
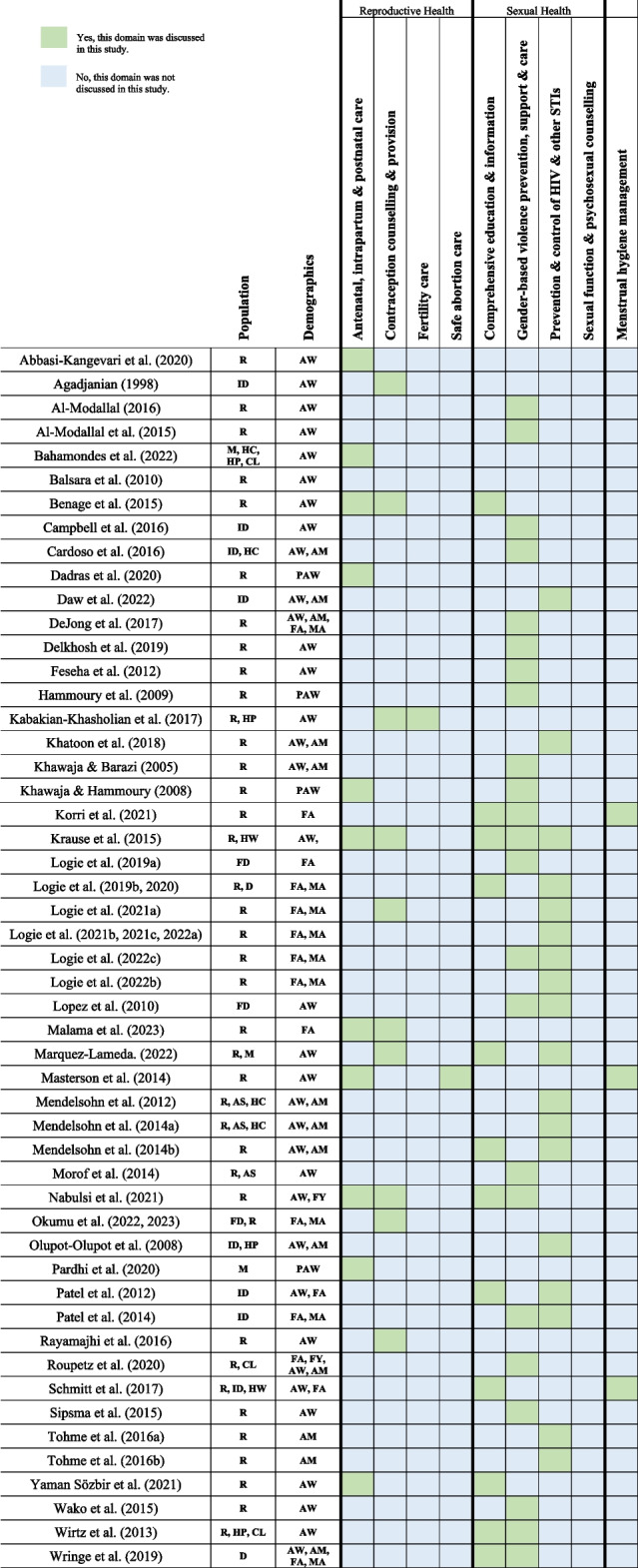
Summary of sexual and reproductive health dimensions examined in included peer-reviewed articles in scoping review of sexual and reproductive health among urban forcibly displaced persons in low and middle-income countries [13, 17, 18, 30–37, 39–75]

*R *refugees, *D *displaced persons, *ID *internally displaced persons, *FD *forcibly displaced persons, *M *migrants, *HW *humanitarian workers, *HP *healthcare providers, *HC *host community members, *CL *community leaders, *AS *asylum seekers, *AW *adult women, *AM *adult men, *PAW *pregnant adult women, *FA *female adolescents, *MAL *male adolescents, *FY *female youth, *MY *male youth

### Sexual and gender-based violence (GBV)

Among the 17 studies that examined GBV [32, 33, 36, 37, 40–42, 45, 46, 52, 61, 68, 69, 73–76] in urban contexts, all explored GBV as it was experienced by women and girls, and one examined experiences of both adolescent boys and girls [52]. Most articles explored experiences of adult women: two explored GBV among adolescent girls [75, 76] and one explored GBV experiences among young women [68].

#### Prevalence and health correlates of intimate partner violence

Of the 17 articles that examined GBV, most (*n* = 11; 64.7%) specifically examined intimate partner violence (IPV) [32, 33, 40–42, 45, 52, 61, 69, 73, 76]. Prevalence ranged from 11.1%-86.0% and varied by age, type of IPV, and external factors. All studies examined the experience of adults, with the exception of two that looked at adolescents, and these found the highest prevalences of IPV at 85.8% and 86.0% [52, 76]. Two articles examined the prevalence of different types of IPV. One study found partner control followed by economic abuse and emotional abuse to be the most common forms of IPV at 73%, 53.3%, and 50.3% respectively [33]. Another study found slapping and throwing objects to be the most common forms of physical IPV [41].

More than half of these articles reported associations between IPV and health and wellbeing (*n* = 6), incuding mental, physical, and other SRH outcomes. For instance, there were associations between experiencing IPV and mental health concerns such as post-traumatic stress disorder symptoms [61] and frequent alcohol use [52]. One study with refugee women in Amman, Irbid and Zarqa, Jordan found an association between psychological IPV and higher rates of health problems including heart, gastrointestinal, liver, respiratory, and urinary problems, recurrent dizziness, fibromyalgia, joint pain, and back pain [32]. Another study with refugee women in Semnan, Iran found IPV exposure was associated with a range of SRH outcomes, including early marriage, sexual coercion, unwanted pregnancy, and a high number of children [40].

The different ways that IPV was measured across studies make it difficult to synthesize these findings, however across studies it appears that a) urban forcibly displaced girls and women are disproportionately exposed to polvictimization (multiple forms of violence); b) there is a range of health challenges linked with IPV exposure, including and extending beyond SRH; and c) married women reported a high prevalence of IPV, including during pregnancy.

#### Risk factors associated with GBV exposure

Seven of the 17 articles that examined GBV explored risks associated with GBV exposure (41.2%) [36, 37, 42, 46, 52, 68, 75]. Three studies collected data from women only [36, 42, 46] while the other four collected data from both women and men [37, 52, 68, 75]. One study found that women were more likely to share stories about sexual harassment while men more likely to discuss other forms of GBV [68].

GBV exposure risks varied across social categories, including age, education, changing social structures and norms, and disruption to social networks and livelihoods. For instance, studies with adolescent girls and young women, including refugees in Beirut, Beqaa, and Tripoli, Lebanon [68] and displaced people in Izmir, Turkey [75], reported that early marriage was associated with risks for further GBV [68, 75]. Among those experiencing early marriage, factors that increased risks for GBV included limited educational opportunities, financial strains, and being alone outside the home [75]. Further, urbanization may change parents’ perspectives on child marriage after arriving in Lebanon, as they may be more likely to view early marriage as a pathway to protecting their daughters and reducing parental responsibility [39].

Among internally displaced adult women, displacement and subsequent loss of social support networks elevated risks for GBV [36, 37]. For instance, in a study conducted in Port-au-Prince, Haiti, destruction of livelihood elevated risks for GBV [36]. Findings paralleled another study in Abidjan, Côte d’Ivoire that documented that poverty, food and housing instability, and changing gender roles and norms increased GBV exposure [37]. Partner characteristics and relationship dynamics were also associated with GBV, including partner alcohol misuse [41, 42]. Among pregnant refugees in Sidon, Lebanon, odds of IPV were higher among those whose husbands did not want the pregnancy [42].

Polyvictimization was also reported [73, 74, 76]. For instance, forcibly displaced women with a history of childhood abuse may be more likely to experience adulthood violence [76], and as adult, forcibly displaced women may report multiple forms (e.g., physical violence, abductions, forced imprisonment, sexual violence, early/forced marriage) and contexts of violence (country of origin, host country) [74]. Together these studies on GBV suggest that multi-level factors, including structural (poverty, livelihood and educational barriers), social (gender inequitable norms, disrupted social networks), and relational (relationship power dynamics, partner alcohol use) level factors increase vulnerability to multiple forms of GBV among urban forcibly displaced persons.

### HIV and other sexually transmitted infections (STIs)

Among the 20 articles examining HIV and STIs, 17 focused only on HIV, one article focused on HIV and transactional sex [50], one on STIs [49], and one on both HIV and STIs [54].

#### HIV and STI testing and prevention

Half of the HIV/STI articles focused on HIV testing and prevention (*n* = 10, 50%) [13, 44, 47, 50, 51, 53, 56, 70, 71, 77]. Most of these were quantitative (*n* = 7) with three qualitative studies. Studies explored experiences among urban forcibly displaced men and women in Uganda [13, 47, 50, 51, 53, 77], Nepal [44], and Peru [56], and refugee men who have sex with men (MSM) in Lebanon [70, 71]. Testing uptake, recorded in six studies, ranged from 29–62% and varied by gender and population [44, 47, 50, 70, 71, 77]. For instance, a study with refugees engaged in transactional sex in Kampala, Uganda found that engaging in transactional sex was associated with lower HIV testing among men, and was not associated with HIV testing among women [50].

Among articles that examined HIV testing [13, 44, 47, 51, 53, 56], transportation costs, overcrowded living conditions, low literacy, and inequitable gender norms were identified as testing barriers [13, 53]. Intersecting stigma—including stigma related to HIV, refugees, sexually active adolescents, and sex workers—also presented barriers to HIV testing among urban refugee youth in Kampala, Uganda [44, 47, 51]. Among urban Venezuelan forcibly displaced women in 6 cities in Peru (Metropolitan Lima, Callao, Tumbes, Cusco, Trujillo, Arequipa), not having health insurance was a barrier to HIV and STI testing [56]. Among MSM in Beirut, Lebanon, lack of comfort with doctors, not seeing a doctor in the past year, and not knowing where to access testing posed as barriers to testing [70, 71] Among forcibly displaced urban refugee youth in Kampala, Uganda, factors associated with STI testing were lower food insecurity and lower adolescent SRH stigma [49].

Several studies focused on HIV vulnerabilities among forcibly displaced persons in urban Uganda [52, 66, 78]. For instance, a study in Gulu with internally displaced men and women reported an HIV prevalence of 12.8%, and risk factors associated with HIV infection included non-consensual sexual debut, past-year STI symptoms, and practicing dry sex (which was defined as sexual intercourse without foreplay or lubrication so that the vagina is dry upon penetration) [66]. Another study in Kampala, Uganda with refugee youth found that depression, alcohol use, and GBV were associated with HIV vulnerabilities, including recent transactional sex and multiple sex partners [52]. There may also be gender differences in HIV vulnerabilities; among urban refugee adolescents in Kampala, Uganda, young men reported higher condom self-efficacy than young women [62, 63]. A study in Beirut, Lebanon found that over half (56.7%) of refugee MSM reported unprotected anal intercourse with men who were HIV positive or did not know their HIV serostatus, and over a third (36%) had engaged in transactional sex [70, 71]. A qualitative study with internally displaced women in Northern Uganda found that the shift away from traditional belief systems, collapse of livelihoods, commuting away from home at night, and inadequate access to SRH information and services elevated HIV vulnerabilities among adolescent girls [78]. Another qualitative study, with forcibly displaced adult women in Medellin, Colombia, found that social and family fragmentation, GBV, abrupt changes in daily lives, and inequitable gender norms elevated HIV and STI acquisition risks [54]. These studies taken together reveal the ways that conflict-related life disruptions (e.g., belief systems, livelihoods, social networks), alongside structural factors (e.g., gender inequities, SGBV across the lifecourse, barriers to accessing SRH services) and relational factors (e.g., sexual practices, low condom efficacy), may increase exposure to HIV and STIs and reduce access to testing.

#### HIV treatment and care

Four articles focused on antiretroviral therapy (ART) and HIV care among urban refugee adult men and women [58–60, 64]. Two quantitative studies in Kuala Lumpur, Malaysia that compared HIV treatment and clinical outcomes between refugees, displaced people, asylum seekers, and host community members found no differences in viral suppression among groups [58, 59]. Qualitative studies explored challenges associated with achieving optimal treatment adherence [60, 64]. One of these studies that included forcibly displaced persons in Kuala Lumpur, Malaysia found that limited access to food, pharmacy stock-outs, and difficulty navigating a new health system were barriers to optimal treatment adherence [60]. The few studies on HIV treatment and care that were included in this review span wide-ranging contexts, presenting challenges in drawing conclusions from this evidence-base and signal the need for more research with urban forcibly displaced persons living with HIV.

### Antenatal care, postnatal care, and contraception

Among the 13 articles that explored antenatal and postnatal care and contraception, six focused on antenatal and postnatal care (46.2%) [30, 34, 35, 38, 65, 72] and seven on contraception and family planning (53.8%) [31, 43, 55, 56, 62, 63, 67]. Most of these studies were conducted with adult forcibly displaced women (*n* = 9); one was conducted with healthcare workers and policy makers alongside adult women [34]. The remaining three studies were conducted with forcibly displaced adolescents, one of which explored experiences of only women [55].

#### Antenatal and postnatal care

Two of the 13 articles that examined antenatal and postnatal care used quantitative methods to explore uptake of antenatal care [30, 35]. One study found that 82.9% of pregnant refugees had received some antenatal care in 14 high refugee density sites, including Beirut, in Lebanon [35], while another study found that pregnant refugees in Tehran, Iran attended an average of 3.73 out of 8 possible antenatal appointments [30]. Four articles explored barriers to accessing care and related risks [34, 38, 65, 72]. One of these studies with pregnant refugees in South Tehran, Iran found that financial constraints, lack of health insurance, transportation challenges, stigma, cultural concerns, legal and immigration issues, and healthcare staff behaviour presented barriers to utilizing prenatal services [38]. Moreover, an article with pregnant forced migrant mothers in Mumbai, India reported that they could not access the antenatal care they need due to unfamiliarity with the local context and a lack of knowledge regarding where to access antenatal care, putting them at a greater risk for poor health outcomes [65]. From these limited studies, structural level challenges (e.g., health insurance barriers, healthcare mistreatment, immigration issues) alongside socio-cultural challenges (e.g., stigma, cultural and religious concerns) posed barriers to antenatal and postnatal care.

#### Contraception

Among the seven articles that explored family planning, five used quantitative methods to explore the access and utilization of contraceptives [55, 56, 62, 63, 67]. One study found that only 20.2% of migrant and refugee women in six urban cities in Peru had access to modern contraceptives [56]. Contraceptive access was reported to be influenced by family and relationship status as well as dynamics. For instance, among migrant and refugee women in six urban sites in Peru, lower socio-economic status was associated with reduced likelihood of emergency contraceptive use, and those who were married or lived with a partner were more likely to use modern and emergency contraceptives [56]. A qualitative study with forcibly displaced women in West Bekaa, Lebanon described that beliefs about wanting a large family size were often in tension with the financial hardships they experienced in displacement, men held the dominant role in making decisions about family planning, and contraceptive access was hindered by the unaffordability of the privatised health system [43]. Another qualitative study found that internally displaced women in Maputo, Mozambique experienced social isolation excluding them from the contraceptive revolution in their host community [31]. Together these studies paint a complex picture of contraceptive access and needs, where some factors associated with low contraception uptake may include structural barriers (e.g., low socio-economic status), relational factors, (e.g., relationship status), and socio-cultural values and priorities (e.g., wishes for larger family sizes) shaped by community norms and experiences of conflict.

### Grey literature findings

Among the 37 included grey literature reports, over three-quarters (*n* = 29) examined GBV [79–108]; these studies are detailed in Table 3. Emergent GBV themes centered on vulnerabilities to experiencing sexual, physical, and psychological abuse. Reports describe forcibly displaced persons in urban humanitarian contexts were at elevated risk for GBV exposure due to various social, cultural, and political dynamics, such as income insecurity, overcrowded living conditions, inequitable gender dynamics, inequitable power dynamics with administrative authorities, and limited awareness of rights [86, 88, 90–94, 97, 101, 104, 105, 108]. Perpetrators of GBV included landlords, neighbors and employers, all of whom displaced people may be dependent on, and in lower positions of power [104, 105]. The main reported targets of violence were women, sexual minorities, and transgender people [83, 85–87, 94–96, 100, 107, 108]. These reports, taken together, emphasize the importance of integrated policies, research, and SRH services to reduce GBV and promote health equity among individuals at risk, including sexually and gender diverse persons. Additionally, the reports emphasize the critical need for support services to aid GBV survivors [79, 82, 85, 98, 103].

**Table 3:** Overview of grey literature articles (*n* = 37) included in scoping review on sexual and reproductive health of urban forcibly displaced persons in low and middle-income countries

Organization (year)	Region	Population	SRH focus	Objective
Care Jordan (2013) [93]	Irbid, Madaba, Mufraq and Zarqa, Jordan	Syrian urban refugees	SGBV/IPV	An assessment of the condition of Syrian refugees in urban areas
Humanitarian Policy Group (2010)	Nairobi, Kenya	Urban refugees	SGBV/IPV	An exploratory review to understand the specific challenges of this community and examine the policy framework and assistance available to them
Humanitarian Policy Group (2011)	Nairobi, Kenya	Internally displaced persons	SGBV/IPV	To understand drivers for displacement, review policy frameworks for IDPs, and identify how the international community can aid
Humanitarian Practice Network (2004) [82]	N/A	Conflict-afflicted peoples	General SRH	To describe advances in policy and what is known about reproductive health needs to equip humanitarian practitioners with essential information for delivering effective reproductive health services to people in crises
International Rescue Committee (2013) [90]	Jordan	Syrian refugees	SGBV/IPV	To assess responses for Syrian refugees as they relate to off-camp and urban programming to understand their conditions and improve program design
MOSAIC and Gender Justice & Society (2020)	Syria, Lebanon, and Turkey	Refugees and internally displaced persons	SGBV/IPV	To understand the impacts of conflict and displacement resulting from the Syrian civil war on LGBTIQ + persons
Norwegian Refugee Council and The Liaison Office (2015)	Jalalabad, Kabul and Kandahar, Afghanistan	Urban internally displaced persons	SGBV/IPV	To assess the needs and vulnerabilities of displaced urban young women and girls in Afghanistan to inform programming
Physicians for Human Rights (2020)	Cox's Bazaar, Bangladesh	Refugees	SGBV/IPV	To corroborate accounts of sexual violence reported as part of the 2017 attacks on the Rohingya with new data from health care workers and describe the physical and mental health needs of Rohingya survivors and the availability of services to meet these needs
Reproductive Health for Urban Refugees Initiative Forced Migration and Refugees Study (FMRS) Program The American University in Cairo (2003)	Cairo, Egypt	Refugees from Sierra Leone and Liberia	SRH literacy	To describe an action research project designed to provide urban refugees with relevant information pertaining to health issues they may face and problem-solving strategies
UNHCR (2004) [83]	N/A	Refugees and internally displaced persons	General SRH	To use the framework for implementation outlined in the Inter-agency Field Manual of Reproductive Health in Refugee Situations, to evaluate reproductive health services to refugees and internally displaced persons
UNHCR (2009) [101]	Nairobi, Kenya	Urban refugees	SGBV/IPV	To document concerns and problems shared by the urban refugee communities regarding sexual and gender-based violence
UNCHR (2009) [101]	N/A	Urban refugees	General SRH	To outline health, education, and livelihood challenges experienced by urban refugees and discuss interventions
UNHCR (2012) [109]	Gulu District, Uganda Pacific Coast, Thailand Mae Sot, Columbia	Adolescent girls in humanitarian settings	General SRH	To describe case studies of adolescent sexual and reproductive health programs assessed to inform services in humanitarian settings
UNCHR (2013)	Nairobi, Kenya	Asylum seekers	SGBV/IPV	To examine the vulnerabilities of asylum seekers and protection concerns immediately after arrival in Nairobi
UNHCR (2016) [110]	Lebanon	Syrian refugees	SRH literacy	To monitor access to and use of key health services among Syrian refugees in Lebanon
United Nations Population Fund (1999) [84]	Albania and Macedonia	Refugee women from Kosovo	SGBV/IPV	To assess the prevalence and experiences of sexual violence among refugees from Kosovo and propose a plan of action to care for the victims
Women's Refugee Commission (2009)	Cairo, Egypt	Refugees and asylum seekers	SGBV/IPV	To develop recommendations to improve the livelihood and decrease vulnerability to GBV for refugee women in Cairo
Women's Refugee Commission (2011) [89]	Johannesburg, South Africa	Refugees, asylum seekers and other refugee-like circumstances	SGBV/IPV	Field assessment of refugees' economic coping strategies as well as related protection risks and market opportunities
Women’s Refugee Commission (2014) [111]	Kampala, Uganda	Refugees with disabilities	General SRH	To understand the needs, vulnerabilities and capacities of refugees with disabilities
Women’s Refugee Commission (2016) [102]	Quito, Ecuador Beirut, Lebanon Kampala, Uganda Delhi, India	Refugees	SGBV/IPV	To determine the nature of GBV risks and humanitarian response that can support refugees in urban settings in 2015
Women's Refugee Commission (2016) [102]	Delhi, India	Urban refugees	SGBV/IPV	To evaluate the community led GBV prevention and response task force operating in refugee communities in Delhi
Women’s Refugee Commission (2017) [103]	Kampala, Uganda Delhi, India Beirut, Lebanon Santo Domingo, Ecuador	Urban refugees	SGBV/IPV	To examine the organisations that Women's Refugee Commission (WRC) partnered with in 2016 to address refugee needs and capabilities in urban settings with an emphasis on GBV
Women’s Refugee Commission (2017) [103]	Kampala and Nakivale Settlement, Uganda	Refugees	Sex work	To outline interventions in Uganda that sought to train refugee women engaged in sex work to be peer educators and bringing mobile clinics to these regions
Women’s Refugee Commission (2017) [103]	Beirut, Lebanon	Transwomen refugees	SGBV/IPV	To outlines a project for transgender women refugees by presenting case studies that analyse GBV risks and response strategies
Women's Refugee Commission and Reproductive Health Uganda (2016)	Kampala, Uganda	Refugees	SGBV/IPV	To address information, service, and support gaps affecting at-risk refugee populations living in Kampala
**Forced Migration Review**				
**Author(s) (year)**	**Region**	**Population**	**SRH focus**	**Objective**
Bray-Watkins (2019) [98]	Central African Republic	Internally displaced persons	SGBV/IPV	To understand sexual coercion and abuse in education settings for displaced children
Chynoweth and Martin (2019)	Kenya, Bangladesh and Italy	Refugee men and boys	SGBV/IPV	To understand sexual violence against men and boys in humanitarian settings
Croome and Hussein (2020) [94]	Somalia/Somaliland	Internally displaced persons	SGBV/IPV	To determine how climate shocks have altered cultural norms and gender dynamics in Somali society
Jaffer, Guy and Niewczasinski (2004) [112]	Yemen	Somali refugees	General SRH	To outline programming and interventions by Marie Stopes International in Yemen to increase sexual and reproductive health services
Kagwanja (2000) [100]	Kenya	Refugees	SGBV/IPV	Examination of the discriminatory nature of Kenyan refugee policy, including its administration and practice, and its role in enabling sexual violence against women refugees
Linn (2020) [95]	Lebanon and Jordan	Refugees	SGBV/IPV	To investigate Syrian refugees' experiences with mobility, security and public space and its relationship with gender
Popinchalk (2008) [113]	Egypt	Refugees	HIV	To determine the accessibility to medical care for HIV-positive refugees
Quintero and Culler (2009) [114]	Columbia	Internally displaced persons	General SRH	To understand the circumstance of displaced Columbians as it relates to health services as well as their unique health challenges
Some (2008) [99]	Kenya	Internally displaced persons	SGBV/IPV	To provide an overview of three assessments investigating rape and sexual abuse among women/girls displaced by the post-election crisis
Sanchez and Enriquez (2004) [115]	Columbia	Internally displaced persons	General SRH	An overview of a project that sought to provide displaced persons with sexual and reproductive health services in the process of restoring their lost citizenship rights
Wells and Kuttiparambil (2020)	Jordan	Syrian refugees	Women’s empowerment	Outline the humanitarian responses targeting women and girls to encourage women to claim more political and social space
Zapata (2020) [96]	San Pedro Sula, Honduras	Internally displaced persons	SGBV/IPV	To understand the outcome of interventions in San Pedro Sula, which provided information about working in high-risk urban neighbourhoods and communities that are affected by various kinds of violence

Other themes identified from the grey literature include sex work, disability, contraception needs, and the needs of people living with HIV. Two reports addressed sex work among displaced people who may fear social and legal consequences (including stigma and prosecution) if their sex work was disclosed; accordingly, mobile clinics were suggested as an appropriate entry point for SRH services tailored for forcibly displaced sex workers [80, 109]. Another report described barriers to accessing SRH services, including HIV/STI testing and family planning, for forcibly displaced persons with disabilities, noting stigma faced by forcibly displaced people with disabilities [106]. Multiple studies described SRH service gaps, notably a lack of choice regarding a variety of family planning methods for forcibly displaced women, and limited access to HIV care for forcibly displaced people living with HIV [113, 115, 116]. Recommendations for improving access to SRH services for urban forcibly displaced people included: (1) improved collaboration between various systems and authorities that forcibly displaced people interface with; (2) wider dissemination of SRH knowledge to forcibly displaced persons; (3) the need to create safe, inclusive, and culturally-aware SRH spaces; and (4) the importance of empowering women and girls in humanitarian contexts to mitigate gender inequity as a barrier to SRH access [110–112, 114].

## Discussion

**Fig. 2:**
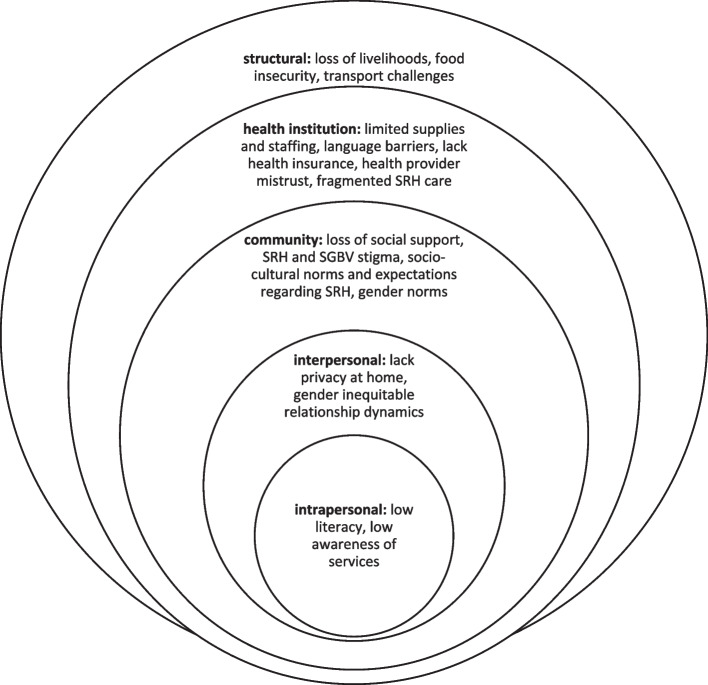
Conceptual framework of multi-level sexual and reproductive health care barriers and challenges among urban forcibly displaced persons in low and middle-income countries

Findings from this scoping review underscore that forcibly displaced individuals in urban LMIC settings face multiple barriers to SRH. These barriers encompass *structural* (e.g., loss of livelihoods, lack of health insurance), *social* (e.g., limited access to community support), *interpersonal* (e.g., gender inequitable relationship dynamics), and *intrapersonal* (e.g., poor mental health) factors. These barriers align with a social ecological [117, 118] approach to health that accounts for the complex interplay between different spheres of influence, and can inform tailored interventions that target one or more levels for change (see Fig. 2). Our findings also identify understudied sexual health (i.e., sexual function and psychosexual counseling) and reproductive health (i.e., fertility care, safe abortion care) domains with this population.

We found across included studies that displacement processes were discussed as exacerbating SRH vulnerabilities among forcibly displaced persons in urban LMIC settings [31, 36, 37, 54, 57, 60, 65, 78]. These included the role of displacement in the breakdown of social support networks and loss of livelihoods in increasing exposure to GBV while also reducing access to sexual health services such as HIV/STI testing. However, the paucity of studies precludes synthesizing experiences by SRH domain (e.g., safe abortion), setting (e.g., slums/informal settlement), or population (e.g., adolescent). A similar limitation was identified by Singh et al. in their 2018 systematic review on the utilization of SRH services in humanitarian crises at large [119]. This observation signals a persistent lack of substantial progress in advancing the field as a whole, and in turn the contextually specific needs of urban forcibly displaced persons. We also found a limited focus on safe abortion and STIs beyond HIV. This suggests a need for additional attention to these understudied SRH issues.

Our findings indicate that stigma experienced by urban forcibly displaced persons presents barriers to SRH prevention, access, and care. Stigma is intersectional, targeting various identities such as refugee status and gender and spans across social-ecological levels, including being manifested at structural (e.g., laws and policies), health institution (e.g., healthcare mistreatment), community (e.g., stigma toward refugees), interpersonal (e.g. gender-based stigma), and intrapersonal (e.g., self-stigma) levels. Moreover, stigma is rooted in drivers and facilitators that could be effectively addressed through targeted stigma-reduction interventions [120]. Stigma within healthcare facilities can reinforce a wider mistrust of health systems among refugee and displaced persons [17, 51]. There is scarcity of SRH interventions focused on stigma reduction with this population.

We documented that resource scarcities (e.g., food, housing, economic) were associated with worse SRH outcomes among urban forcibly displaced persons [37, 60, 75, 76]. This reflects the long-standing insufficient funding and resources for SRH (and health care more generally) in humanitarian settings [48]. Once a forcibly displaced person leaves a formal refugee settlement/camp to migrate to urban regions, many forgo formal financial support offered by UNHCR or other refugee settlement-based organizations to refugees living in settlements, such as food, land/housing, or economic stipends. They may then experience financial challenges, such as transportation costs to accessing healthcare, high rent in cities and/or substandard housing in urban informal settlements, in addition to lack of health insurance in some contexts. These resource scarcity barriers to SRH care are further exacerbated by individual-level barriers such as low literacy and language barriers, and systemic-level barriers such as insufficient staffing and medication stock-outs.

**Fig. 3:**
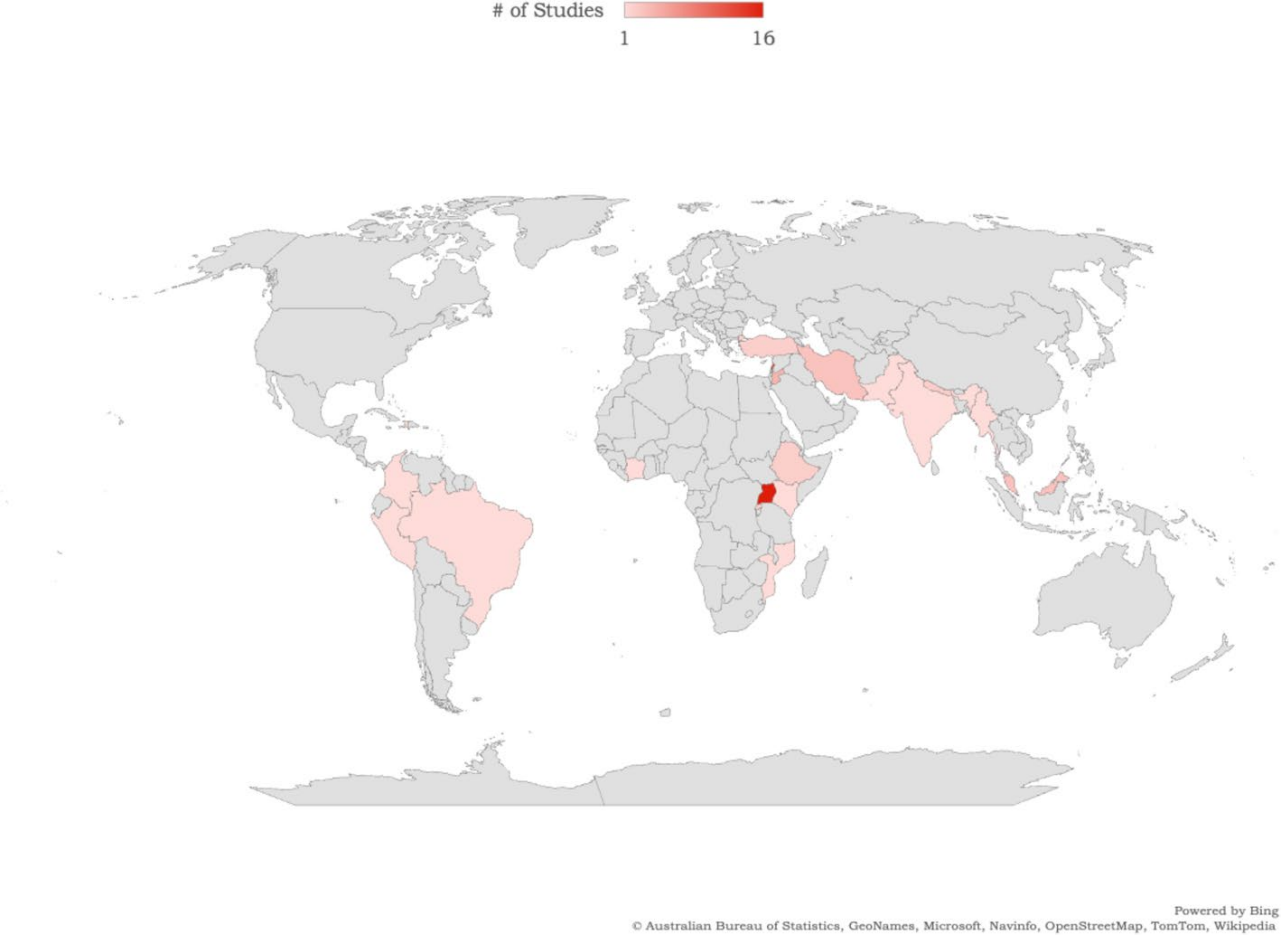
Map of countries of included peer-reviewed studies in this scoping review of urban forcibly displaced persons' sexual and reproductive health in low and middle-income countries. Included countries are represented with colours reflecting the number of studies from each country reported in the figure legend

**Fig. 4:**
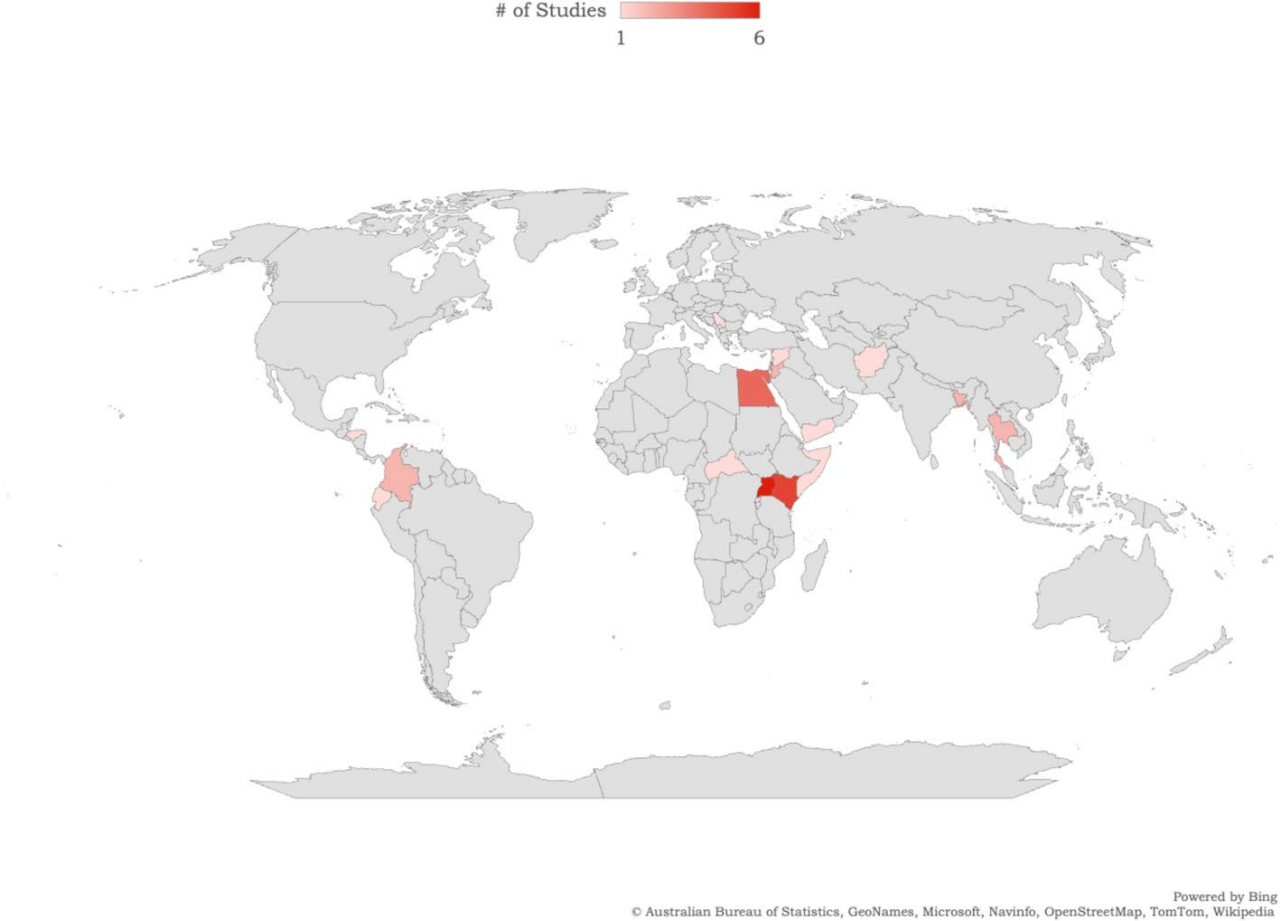
Map of countries of included grey literature studies in this scoping review of urban forcibly displaced persons' sexual and reproductive health in low and middle-income countries. Included countries are represented with colours reflecting the number of studies from each country reported in the figure legend

Our study has limitations. We focused on a select range of SRH outcomes as defined by a SRH conceptual framework [28, 29] and may have overlooked other important issues relevant to SRH outside of this (e.g., fistulae). Our criteria for language inclusion may have omitted some relevant articles. As there was so many different contexts, article types, refugee types (e.g., displaced, refugee), and populations (e.g., adolescents, pregnant adult women), we could not conduct a meta-analysis, and even when synthesizing key findings this heterogeneity presented challenges in contextualizing SRH findings within each setting and its socio-cultural norms, geography, country income, and laws and other social determinants of health. It is plausible that urban refugees may share health status outcomes with host communities while living in urban informal settlements or slums due to the nature of shared socio-cultural and economic conditions in slums [121], yet these similarities and/or differences in SRH outcomes with host communities were beyond the scope of this review. Further, the studies included in our analysis exhibited a significant underrepresentation of large global regions, namely Africa, Latin America, and the Caribbean. This limited inclusion of studies from these regions hampers our understanding of the specific needs and priorities of urban forcibly displaced persons residing in these urban contexts (Figs. 3 and 4). Despite these limitations, this review’s strengths include its unique focus on urban forcibly displaced persons in LMIC contexts, where the majority of forcibly displaced persons live. Our review also reinforces the need to include multiply marginalized communities in future SRH research—including urban forcibly displaced sex workers, people who use drugs, and lesbian, gay, bisexual, and transgender persons [122–124].

Urgent research and interventions are needed to address SRH challenges faced by urban forcibly displaced persons; these strategies can ultimately advance health equity and well-being not only for forcibly displaced persons, but in the case of those living in slums, interventions may have multiplier effects [121]. Future research can identify targets for stigma reduction (e.g., healthcare workers, refugee women) and implement evidence-based intersectional stigma reduction strategies to mitigate barriers to accessing SRH care [125]. Effectively advancing SRH in humanitarian settings requires resources for implementing and evaluating multi-level interventions integrated within existing health systems, as well as community-level, family-level, and individual-level approaches. Such interventions can specifically address health literacy and language needs of urban forcibly displaced persons, transportation-related challenges (e.g., via mobile clinics), and, when needed, extend health insurance coverage to forcibly displaced individuals. Additionally, innovative approaches such as self-care strategies for SRH (e.g., HIV self-testing, long-acting self-injectable contraception, over-the-counter oral contraception, abortion self-management) hold significant promise in addressing some of these aforementioned SRH barriers and can be explored and tested with urban forcibly displaced persons. These self-care strategies may help to overcome challenges related to privacy, transportation, and healthcare provider mistrust [48, 126], yet they also require an enabling social and health environment, so can be offered in tandem with strategies focused on advancing social and health equity [126].

## Conclusion

This review identified barriers to SRH care spanning social-ecological levels [117, 118] among urban forcibly displaced persons in LMIC contexts. The process of displacement, resource insecurity, and stigma exacerbate and drive SRH vulnerabilities for urban forcibly displaced persons in LMIC contexts. However, there remain critical knowledge gaps regarding a range of SRH issues across diverse LMIC settings, with particular knowledge gaps regarding socially marginalized populations. Our findings signal that in urban LMIC settings, there may be unique barriers to accessing SRH information, resources and care faced by forcibly displaced persons (e.g., no financial support from UNHCR or other refugee agencies, social isolation, language barriers at clinics) compared to formal refugee settlements where persons may have more access to refugee communities, translators at clinics, and financial stipends (e.g., housing, land, food supplements). Future research and action are required to address the unique and often unmet SRH needs among urban forcibly displaced persons to advance health and rights.

### Supplementary Information

**Supplementary Material 1.**

## Data Availability

The datasets used and/or analysed during the current study available from the corresponding author on reasonable request.
