# Supplementary material for: Sexual and reproductive health among forcibly displaced persons in urban environments in low and middle-income countries: scoping review findings
Source: Reprod Health. 2024 Apr 12;21:51. doi: 10.1186/s12978-024-01780-7 (PMC11010352; doi:10.1186/s12978-024-01780-7)
Supplement: Supplementary file 1 — Supplementary Material 1. [file 12978_2024_1780_MOESM1_ESM.docx]

**Supplementary File 1. Primary search strategy for ‘Sexual and reproductive health among forcibly displaced persons in urban environments in low and middle-income countries: scoping review findings’**

**Database:** MEDLINE

| **Search #** | **Terms searched** |
| --- | --- |
| 1 | exp Hospitals, Urban/ OR exp Urban Health/ OR exp Urban Health Services/ OR exp Urban Population/ OR exp Cities/ OR (urban or cities or city or municipal*).tw,kf. |
| 2 | exp Refugees/ OR (refugee* or displace* or asylum or migrant).tw,kf. |
| 3 | ((urban or cities or city or municipal*) adj3 (refugee* or displace* or asylum or migrant)).tw,kf. |
| 4 | 1 and 2 |
| 5 | 4 or 3 |
| 6 | exp Sexual Health/ OR (sexual health*).tw,kf. OR (sexual adj3 health*).tw,kf. |
| 7 | Domestic Violence/ or (domestic adj3 (abus* or violen*)).tw,kf. |
| 8 | exp Intimate Partner Violence/ or ((partner* or spous* or wife or wives) adj3 (abus* or violen*)).tw,kf |
| 9 | Gender-Based Violence/ or gender-based violence.tw,kf. |
| 10 | Rape/ or rape.tw,kf. |
| 11 | Sex Offenses/ or Sex Education/ or Sex Counseling/ or (sex* adj3 (abus* or offen*or health or educat* or counsel*)).tw,kf. or family planning.tw,kf |
| 12 | exp Sexually Transmitted Diseases/ or ((sexually transmitted or venereal) adj3 (diseas* or infect*)).tw,kf. or (sti or stis).tw,kf. or (std or stds).tw,kf. |
| 13 | exp HIV Infections/ or (HIV adj3 infect*).tw,kf. |
| 14 | exp Syphilis/ or syphilis.tw,kf. |
| 15 | exp Chlamydia/ or exp Chlamydia Infections/ or chlamydia.tw,kf. |
| 16 | Gonorrhea/ or gonorrhea.tw,kf. |
| 17 | 6 or 7 or 8 or 9 or 10 or 11 or 12 or 13 or 14 or 15 or 16 |
| 18 | (Afghanistan or Guinea Bissau or Sierra Leone or Somalia or Burkina Faso or Democratic People's Republic of Korea or South Sudan or Burundi or Liberia or Syrian Arab Republic or Central African Republic or Madagascar or Chad or Malawi or Tanzania or Comoros or Mali or Togo or Democratic Republic of Congo or Mozambique or Uganda or Eritrea or Nepal or Republic of Yemen or Ethiopia or Niger or Zimbabwe or Gambia or Rwanda or Guinea or Senegal or Sudan).tw,kf. |
| 19 | (((((Angola or Papua New Guinea or Bangladesh or Kenya or Philippines or Bhutan or Kiribati or Sao Tome) and Principe) or Bolivia or Solomon Islands or Cabo Verde or Kyrgyz Republic or Sri Lanka or Cambodia or Laos or Lao PDR or Cameroon or Lesotho or Republic of Congo or Mauritania or Timor-Leste or Cote dIvoire or Tunisia or Djibouti or Ukraine or Egypt or Mongolia or Uzbekistan or El Salvador or Morocco or Vanuatu or Myanmar or Vietnam or Ghana or Nicaragua or West Bank) and Gaza) or Honduras or Nigeria or Zambia or India or Pakistan or Algeria or Benin or Senegal Republic or Tanzania or Micronesia or Comoros or Nepal or Ewastini or Zimbabwe or Palestine or Iran or Indonesia or Haiti or Tajikistan or Belize or Samoa).tw,kf. |
| 20 | (((((Albania or Fiji or Namibia or Gabon or American Samoa or Grenada or Paraguay or Armenia or Guatemala or Peru or Azerbaijan or Guyana or Romania or Belarus or Russian Federation or Iraq or Bosnia) and Herzegovina) or Jamaica or Serbia or Botswana or Jordan or South Africa or Brazil or Kazakhstan or St Lucia or Bulgaria or Lebanon or St Vincent) and the Grenadines) or China or Libya or Suriname or Colombia or Macedonia or Thailand or Costa Rica or Malaysia or Tonga or Cuba or Maldives or Turkey or Dominica or Marshall Islands or Turkmenistan or Dominican Republic or Mauritius or Tuvalu or Equatorial Guinea or Mexico or Venezuela or Ecuador or Montenegro or Argentina or Georgia or Kosovo or Moldova or Panama).tw,kf. |
| 21 | Developing Nations/ or Least Developed Countries/ or Less-Developed Countries/ or Less-Developed Nations/ or Third-World Countries/ or Third-World Nations/ or Under-Developed Countries/ or Under-Developed Nations/ |
| 22 | (((Low income countries or Middle income countries or Low) and middle income countries) or Global south or Least developed countries or Under developed countries or Third world countries or Second world countries or Resource limited countries).tw,kf. |
| 23 | 18 or 19 or 20 or 21 or 22 |
| 24 | Exp Contraception/ OR Reproductive Health/ OR exp Prenatal Care/ OR exp Pregnancy/ OR exp Postnatal Care/ OR exp Abortion, Habitual/ or exp Abortion, Spontaneous/ or exp Abortion, Eugenic/ or exp Abortion, Legal/ or exp Abortion, Induced/ OR exp Fertility/ or exp Fertility Clinics/ OR Menstruation/ |
| 25 | (contracepti* or abortion or fertility or antenatal or intrapartum or postnatal or menstru*).tw,kf. |
| 26 | 24 or 25 |
| 27 | 17 or 26 |
| 28 | 5 and 23 and 27 |

**Database:** EMBASE

| **Search #** | **Terms searched** |
| --- | --- |
| 1 | urban population/ OR urban health/ OR urban hospital/ OR city/ OR (urban or cities or city or municipal*).tw,kw. |
| 2 | exp refugee/ OR exp refugee camp/ OR (refugee* or displace* or asylum or migrant).tw,kw. |
| 3 | ((urban or cities or city or municipal*) adj3 (refugee* or displace* or asylum or migrant)).tw,kw. |
| 4 | 1 and 2 |
| 5 | 4 or 3 |
| 6 | exp sexual health/ OR (sexual health*).tw,kw. OR (sexual adj3 health*).tw,kw. |
| 7 | exp domestic violence/ or (domestic adj3 (abus* or violen*)).tw,kw. |
| 8 | exp partner violence/ OR battered women/ or ((partner* or spous* or wife or wives) adj3 (abus* or violen*)).tw,kw |
| 9 | gender based violence/ or gender-based violence.tw,kw. |
| 10 | rape/ or rape.tw,kw. |
| 11 | sexual crime/ or sexual education/ or sexual counseling/ or family planning/ or (sex* adj3 (abus* or offen*or health or educat* or counsel*)).tw,kw. or family planning.tw,kw |
| 12 | exp sexually transmitted diseases/ or ((sexually transmitted or venereal) adj3 (diseas* or infect*)).tw,kw. or (sti or stis).tw,kw. or (std or stds).tw,kw. |
| 13 | exp Human immunodeficiency virus/ or (HIV adj3 infect*).tw,kw. |
| 14 | exp syphilis/ or syphilis.tw,kw. |
| 15 | exp Chlamydia/ or exp Chlamydia Infections/ or chlamydia.tw,kw. |
| 16 | gonorrhea/ or gonorrhea.tw,kw. |
| 17 | 6 or 7 or 8 or 9 or 10 or 11 or 12 or 13 or 14 or 15 or 16 |
| 18 | (Afghanistan or Guinea Bissau or Sierra Leone or Somalia or Burkina Faso or Democratic People's Republic of Korea or South Sudan or Burundi or Liberia or Syrian Arab Republic or Central African Republic or Madagascar or Chad or Malawi or Tanzania or Comoros or Mali or Togo or Democratic Republic of Congo or Mozambique or Uganda or Eritrea or Nepal or Republic of Yemen or Ethiopia or Niger or Zimbabwe or Gambia or Rwanda or Guinea or Senegal or Sudan).tw,kw. |
| 19 | (((((Angola or Papua New Guinea or Bangladesh or Kenya or Philippines or Bhutan or Kiribati or Sao Tome) and Principe) or Bolivia or Solomon Islands or Cabo Verde or Kyrgyz Republic or Sri Lanka or Cambodia or Laos or Lao PDR or Cameroon or Lesotho or Republic of Congo or Mauritania or Timor-Leste or Cote dIvoire or Tunisia or Djibouti or Ukraine or Egypt or Mongolia or Uzbekistan or El Salvador or Morocco or Vanuatu or Myanmar or Vietnam or Ghana or Nicaragua or West Bank) and Gaza) or Honduras or Nigeria or Zambia or India or Pakistan or Algeria or Benin or Senegal Republic or Tanzania or Micronesia or Comoros or Nepal or Ewastini or Zimbabwe or Palestine or Iran or Indonesia or Haiti or Tajikistan or Belize or Samoa).tw,kw. |
| 20 | (((((Albania or Fiji or Namibia or Gabon or American Samoa or Grenada or Paraguay or Armenia or Guatemala or Peru or Azerbaijan or Guyana or Romania or Belarus or Russian Federation or Iraq or Bosnia) and Herzegovina) or Jamaica or Serbia or Botswana or Jordan or South Africa or Brazil or Kazakhstan or St Lucia or Bulgaria or Lebanon or St Vincent) and the Grenadines) or China or Libya or Suriname or Colombia or Macedonia or Thailand or Costa Rica or Malaysia or Tonga or Cuba or Maldives or Turkey or Dominica or Marshall Islands or Turkmenistan or Dominican Republic or Mauritius or Tuvalu or Equatorial Guinea or Mexico or Venezuela or Ecuador or Montenegro or Argentina or Georgia or Kosovo or Moldova or Panama).tw,kw. |
| 21 | exp developing country |
| 22 | (((Low income countries or Middle income countries or Low) and middle income countries) or Global south or Least developed countries or Under developed countries or Third world countries or Second world countries or Resource limited countries).tw,kw. |
| 23 | 18 or 19 or 20 or 21 or 22 |
| 24 | exp contraception/ OR exp spontaneous abortion/ OR exp abortion/ OR exp legal abortion/ or exp abortion/ or exp recurrent abortion/ or exp induced abortion/ or exp medical abortion/ OR exp fertility/ or exp fertility clinic/ OR exp prenatal care/ or exp pregnancy/ OR exp menstruation/ OR exp reproductive health/ |
| 25 | (contracepti* or abortion or fertility or antenatal or intrapartum or postnatal or menstru*).tw,kw. |
| 26 | 24 or 25 |
| 27 | 26 or 17 |
| 24 | 5 and 23 and 27 |

**Database:** PsychInfo

| **Search #** | **Terms searched** |
| --- | --- |
| 1 | exp Urban Health/ OR exp Urban Environments/ OR (urban or cities or city or municipal*).tw. |
| 2 | exp Refugees/ OR Human Migration/ OR Asylum Seeking OR Political Asylum/ OR (refugee* or displace* or asylum or migrant).tw. |
| 3 | ((urban or cities or city or municipal*) adj3 (refugee* or displace* or asylum or migrant)).tw. |
| 4 | 1 and 2 |
| 5 | 4 or 3 |
| 6 | exp Sexual Health/ OR (sexual health*).tw. OR (sexual adj3 health*).tw. |
| 7 | Domestic Violence/ or (domestic adj3 (abus* or violen*)).tw. |
| 8 | exp Intimate Partner Violence/ or ((partner* or spous* or wife or wives) adj3 (abus* or violen*)).tw. |
| 9 | gender-based violence.tw. |
| 10 | Rape/ or rape.tw. |
| 11 | Sexual Abuse/ or Sex Offenses/ or Sex Education/ or Sex Therapy/ or Family Planning/ or (sex* adj3 (abus* or offen*or health or educat* or counsel* or therap*)).tw. or family planning.tw. |
| 12 | exp Sexually Transmitted Diseases/ or ((sexually transmitted or venereal) adj3 (diseas* or infect*)).tw. or (sti or stis).tw. or (std or stds).tw. |
| 13 | exp HIV/ or (HIV adj3 infect*).tw. |
| 14 | exp Syphilis/ or syphilis.tw. |
| 15 | chlamydia.tw. |
| 16 | Gonorrhea/ or gonorrhea.tw. |
| 17 | 6 or 7 or 8 or 9 or 10 or 11 or 12 or 13 or 14 or 15 or 16 |
| 18 | (Afghanistan or Guinea Bissau or Sierra Leone or Somalia or Burkina Faso or Democratic People's Republic of Korea or South Sudan or Burundi or Liberia or Syrian Arab Republic or Central African Republic or Madagascar or Chad or Malawi or Tanzania or Comoros or Mali or Togo or Democratic Republic of Congo or Mozambique or Uganda or Eritrea or Nepal or Republic of Yemen or Ethiopia or Niger or Zimbabwe or Gambia or Rwanda or Guinea or Senegal or Sudan).tw. |
| 19 | (((((Angola or Papua New Guinea or Bangladesh or Kenya or Philippines or Bhutan or Kiribati or Sao Tome) and Principe) or Bolivia or Solomon Islands or Cabo Verde or Kyrgyz Republic or Sri Lanka or Cambodia or Laos or Lao PDR or Cameroon or Lesotho or Republic of Congo or Mauritania or Timor-Leste or Cote dIvoire or Tunisia or Djibouti or Ukraine or Egypt or Mongolia or Uzbekistan or El Salvador or Morocco or Vanuatu or Myanmar or Vietnam or Ghana or Nicaragua or West Bank) and Gaza) or Honduras or Nigeria or Zambia or India or Pakistan or Algeria or Benin or Senegal Republic or Tanzania or Micronesia or Comoros or Nepal or Ewastini or Zimbabwe or Palestine or Iran or Indonesia or Haiti or Tajikistan or Belize or Samoa).tw. |
| 20 | (((((Albania or Fiji or Namibia or Gabon or American Samoa or Grenada or Paraguay or Armenia or Guatemala or Peru or Azerbaijan or Guyana or Romania or Belarus or Russian Federation or Iraq or Bosnia) and Herzegovina) or Jamaica or Serbia or Botswana or Jordan or South Africa or Brazil or Kazakhstan or St Lucia or Bulgaria or Lebanon or St Vincent) and the Grenadines) or China or Libya or Suriname or Colombia or Macedonia or Thailand or Costa Rica or Malaysia or Tonga or Cuba or Maldives or Turkey or Dominica or Marshall Islands or Turkmenistan or Dominican Republic or Mauritius or Tuvalu or Equatorial Guinea or Mexico or Venezuela or Ecuador or Montenegro or Argentina or Georgia or Kosovo or Moldova or Panama).tw. |
| 21 | Developing Countries/ |
| 22 | (((Low income countries or Middle income countries or Low) and middle income countries) or Global south or Least developed countries or Under developed countries or Third world countries or Second world countries or Resource limited countries).tw. |
| 23 | 18 or 19 or 20 or 21 or 22 |
| 24 | exp Birth Control/ OR exp Spontaneous Abortion/ OR exp Induced Abortion/ OR exp Fertility/ OR exp Prenatal Care/ or exp Pregnancy/ OR Intrapartum Period/ OR exp Postnatal Period/ OR exp Menstruation/ OR exp Reproductive Health/ |
| 25 | (contracepti* or abortion or fertility or antenatal or intrapartum or postnatal or menstru*).tw. |
| 26 | 24 or 25 |
| 27 | 17 or 26 |
| 24 | 5 and 23 and 27 |

**Database:** CINAHL

| **Search #** | **Terms searched** |
| --- | --- |
| S1 | (MH "Urban Population") OR (MH "Hospitals, Urban") OR (MH "Urban Health Services") OR (MH "Urban Health") OR (MH "Urban Areas") OR TI(urban OR cities OR city OR municipal*) OR AB(urban OR cities OR city OR municipal*) |
| S2 | (MH "Refugee Camps") OR (MH "Refugees+") OR TI(refugee* OR displace* OR asylum OR migrant) OR AB (refugee* OR displace* OR asylum OR migrant) |
| S3 | S2 and S1 |
| S4 | TI((urban OR cities OR city OR municipal*) N3 (refugee* OR displace* OR asylum OR migrant)) OR AB((urban OR cities OR city OR municipal*) N3 (refugee* OR displace* OR asylum OR migrant)) |
| **S5** | S4 or S3 |
| S6 | (MH "Sexual Health") OR TI(sexual health) OR AB(sexual health) OR TI((sexual) N3 (health)) OR AB((sexual) N3 (health)) |
| S7 | (MH "Domestic Violence+") OR (MH "Intimate Partner Violence") OR (MH "Gender-Based Violence") OR (MH "Dating Violence") |
| S8 | TI(domestic N3 (abus* or violen*)) OR AB(domestic N3 (abus* or violen*)) OR TI((partner* or spouse* or wife or wives) N3 (abus* or violen*)) OR AB((partner* or spouse* or wife or wives) N3 (abus* or violen*)) OR TI(gender-based violence) OR AB(gender-based violence) OR TI(dating violence) OR AB(dating violence) |
| S9 | (MH "Rape") OR (MH "Sexual Abuse+") |
| S10 | TI(rape) OR AB(rape) OR TI(sex* N3 (abus* or offen* or health or educat* or counsel*)) OR TI(family planning) OR AB(sex* N3 (abus* or offen* or health or educat* or counsel*)) OR AB(family planning) |
| S11 | (MH “Sex Education”) OR (MH “Sex Counseling) |
| S12 | (MH "Sexually Transmitted Diseases+") OR (MH "Sexually Transmitted Diseases, Bacterial") OR (MH "Sexually Transmitted Diseases, Fungal") OR (MH "Sexually Transmitted Diseases, Protozoal") OR (MH "Sexually Transmitted Diseases, Viral") |
| S13 | TI((sexually transmitted or venereal) N3 (diseas* or infect*)) OR AB((sexually transmitted or venereal) N3 (diseas* or infect*)) OR TI(sti or stis or std or stds) OR AB(sti or stis or std or stds) |
| S14 | (MH "Human Immunodeficiency Virus+") OR (MH "Syphilis") OR (MH "Gonorrhea") OR (MH "Chlamydia") OR (MH "Chlamydia Infections") |
| S15 | TI(HIV N3 infect*) OR AB(HIV N3 infect*) OR TI(syphilis) OR AB(syphilis) OR TI(chlamydia) or AB(chlamydia) OR TI(gonorrhea) OR AB(gonorrhea) |
| **S16** | S6 OR S7 OR S8 OR S9 OR S10 OR S11 OR S12 OR S13 OR S14 OR S15 |
| S17 | TI(Afghanistan or Guinea Bissau or Sierra Leone or Somalia or Burkina Faso or Democratic People's Republic of Korea or South Sudan or Burundi or Liberia or Syrian Arab Republic or Central African Republic or Madagascar or Chad or Malawi or Tanzania or Comoros or Mali or Togo or Democratic Republic of Congo or Mozambique or Uganda or Eritrea or Nepal or Republic of Yemen or Ethiopia or Niger or Zimbabwe or Gambia or Rwanda or Guinea or Senegal or Sudan) OR AB(Afghanistan or Guinea Bissau or Sierra Leone or Somalia or Burkina Faso or Democratic People's Republic of Korea or South Sudan or Burundi or Liberia or Syrian Arab Republic or Central African Republic or Madagascar or Chad or Malawi or Tanzania or Comoros or Mali or Togo or Democratic Republic of Congo or Mozambique or Uganda or Eritrea or Nepal or Republic of Yemen or Ethiopia or Niger or Zimbabwe or Gambia or Rwanda or Guinea or Senegal or Sudan) |
| S18 | TI(((((Angola or Papua New Guinea or Bangladesh or Kenya or Philippines or Bhutan or Kiribati or Sao Tome) and Principe) or Bolivia or Solomon Islands or Cabo Verde or Kyrgyz Republic or Sri Lanka or Cambodia or Laos or Lao PDR or Cameroon or Lesotho or Republic of Congo or Mauritania or Timor-Leste or Cote dIvoire or Tunisia or Djibouti or Ukraine or Egypt or Mongolia or Uzbekistan or El Salvador or Morocco or Vanuatu or Myanmar or Vietnam or Ghana or Nicaragua or West Bank) and Gaza) or Honduras or Nigeria or Zambia or India or Pakistan or Algeria or Benin or Senegal Republic or Tanzania or Micronesia or Comoros or Nepal or Ewastini or Zimbabwe or Palestine or Iran or Indonesia or Haiti or Tajikistan or Belize or Samoa) OR AB(((((Angola or Papua New Guinea or Bangladesh or Kenya or Philippines or Bhutan or Kiribati or Sao Tome) and Principe) or Bolivia or Solomon Islands or Cabo Verde or Kyrgyz Republic or Sri Lanka or Cambodia or Laos or Lao PDR or Cameroon or Lesotho or Republic of Congo or Mauritania or Timor-Leste or Cote dIvoire or Tunisia or Djibouti or Ukraine or Egypt or Mongolia or Uzbekistan or El Salvador or Morocco or Vanuatu or Myanmar or Vietnam or Ghana or Nicaragua or West Bank) and Gaza) or Honduras or Nigeria or Zambia or India or Pakistan or Algeria or Benin or Senegal Republic or Tanzania or Micronesia or Comoros or Nepal or Ewastini or Zimbabwe or Palestine or Iran or Indonesia or Haiti or Tajikistan or Belize or Samoa) |
| S19 | TI(((((Albania or Fiji or Namibia or Gabon or American Samoa or Grenada or Paraguay or Armenia or Guatemala or Peru or Azerbaijan or Guyana or Romania or Belarus or Russian Federation or Iraq or Bosnia) and Herzegovina) or Jamaica or Serbia or Botswana or Jordan or South Africa or Brazil or Kazakhstan or St Lucia or Bulgaria or Lebanon or St Vincent) and the Grenadines) or China or Libya or Suriname or Colombia or Macedonia or Thailand or Costa Rica or Malaysia or Tonga or Cuba or Maldives or Turkey or Dominica or Marshall Islands or Turkmenistan or Dominican Republic or Mauritius or Tuvalu or Equatorial Guinea or Mexico or Venezuela or Ecuador or Montenegro or Argentina or Georgia or Kosovo or Moldova or Panama) OR AB(((((Albania or Fiji or Namibia or Gabon or American Samoa or Grenada or Paraguay or Armenia or Guatemala or Peru or Azerbaijan or Guyana or Romania or Belarus or Russian Federation or Iraq or Bosnia) and Herzegovina) or Jamaica or Serbia or Botswana or Jordan or South Africa or Brazil or Kazakhstan or St Lucia or Bulgaria or Lebanon or St Vincent) and the Grenadines) or China or Libya or Suriname or Colombia or Macedonia or Thailand or Costa Rica or Malaysia or Tonga or Cuba or Maldives or Turkey or Dominica or Marshall Islands or Turkmenistan or Dominican Republic or Mauritius or Tuvalu or Equatorial Guinea or Mexico or Venezuela or Ecuador or Montenegro or Argentina or Georgia or Kosovo or Moldova or Panama) |
| S20 | TI(((Low income countries or Middle income countries or Low) and middle income countries) or Least developed countries or Under developed countries or Third world countries or Second world countries or Resource limited countries) OR AB(((Low income countries or Middle income countries or Low) and middle income countries) or Least developed countries or Under developed countries or Third world countries or Second world countries or Resource limited countries) |
| S21 | (MH "Developing Countries") OR (MH "Low and Middle Income Countries") |
| **S22** | S17 OR S18 OR S19 OR S20 OR S21 |
| **S23** | TI(contracepti* or abortion or fertility or antenatal or intrapartum or postnatal or menstru*) OR AB(contracepti* or abortion or fertility or antenatal or intrapartum or postnatal or menstru*) |
| **S24** | (MH "Abortion, Induced+") OR (MH "Abortion, Spontaneous+") OR (MH “Reproductive Health”) OR (MH “Contraception+”) OR (MH “Fertility+”) OR (MH “Menstruation”) OR (MH “Prenatal Care”) OR (MH “Pregnancy+”) OR (MH “Postnatal Care+”) |
| **S25** | S23 or S24 |
| **S26** | S25 or S16 |
| S23 | S5 AND S22 AND S26 |

**Database:** SSCI

| **Search #** | **Terms Searched** |
| --- | --- |
| 1 | TI=(refugee* or displace* or asylum or migrant) OR AB=(refugee* or displace* or asylum or migrant) |
| 2 | TI=(urban or cities or city or municipal*) OR AB=(urban or cities or city or municipal*) |
| 3 | TI = (“domestic violence” or (domestic NEAR/3 (abus* or violen*))) OR AB=(“domestic violence” or (domestic NEAR/3 (abus* or violen*))) |
| 4 | TI= (“partner violence” or “intimate partner violence” or ((partner* or spous* or wife or wives) NEAR/4 (abus* or violen*))) OR AB= (“partner violence” or “intimate partner violence” or ((partner* or spous* or wife or wives) NEAR/4 (abus* or violen*))) |
| 5 | TI=(“gender based violence”) OR AB=(“gender based violence”) |
| 6 | TI=(Rape) OR AB=(Rape) |
| 7 | TI= ((sex* NEAR/3 abus*) or (sex* NEAR/3 offen*) or (sex* NEAR/3 health) or (sex* NEAR/3 educat*) or (sex* NEAR/3 counsel*) or (sex* NEAR/3 therap*) or family planning) OR AB=((sex* NEAR/3 abus*) or (sex* NEAR/3 offen*) or (sex* NEAR/3 health) or (sex* NEAR/3 educat*) or (sex* NEAR/3 counsel*) or (sex* NEAR/3 therap*) or family planning) |
| 8 | TI= (((“sexually transmitted” or venereal) NEAR/3 (diseas* or infect*))or (sti or stis) or (std or stds)) OR AB=(((“sexually transmitted” or venereal) NEAR/3 (diseas* or infect*))or (sti or stis) or (std or stds)) |
| 9 | TI= (“Human immunodeficiency virus” or (HIV NEAR/3 infect*)) OR AB= (“Human immunodeficiency virus” or (HIV NEAR/3 infect*)) |
| 10 | TI=((Syphilis) or (Chlamydia or “Chlamydia Infections”) or (gonorrhea)) OR AB=((Syphilis) or (Chlamydia or “Chlamydia Infections”) or (gonorrhea)) |
| 11 | TI=((Afghanistan or Guinea Bissau or Sierra Leone or Somalia or Burkina Faso or Democratic People's Republic of Korea or South Sudan or Burundi or Liberia or Syrian Arab Republic or Central African Republic or Madagascar or Chad or Malawi or Tanzania or Comoros or Mali or Togo or Democratic Republic of Congo or Mozambique or Uganda or Eritrea or Nepal or Republic of Yemen or Ethiopia or Niger or Zimbabwe or Gambia or Rwanda or Guinea or Senegal or Sudan) OR (((((Angola or Papua New Guinea or Bangladesh or Kenya or Philippines or Bhutan or Kiribati or Sao Tome) and Principe) or Bolivia or Solomon Islands or Cabo Verde or Kyrgyz Republic or Sri Lanka or Cambodia or Laos or Lao PDR or Cameroon or Lesotho or Republic of Congo or Mauritania or Timor-Leste or Cote dIvoire or Tunisia or Djibouti or Ukraine or Egypt or Mongolia or Uzbekistan or El Salvador or Morocco or Vanuatu or Myanmar or Vietnam or Ghana or Nicaragua or West Bank) and Gaza) or Honduras or Nigeria or Zambia or India or Pakistan or Algeria or Benin or Senegal Republic or Tanzania or Micronesia or Comoros or Nepal or Ewastini or Zimbabwe or Palestine or Iran or Indonesia or Haiti or Tajikistan or Belize or Samoa) OR (((((Albania or Fiji or Namibia or Gabon or American Samoa or Grenada or Paraguay or Armenia or Guatemala or Peru or Azerbaijan or Guyana or Romania or Belarus or Russian Federation or Iraq or Bosnia) and Herzegovina) or Jamaica or Serbia or Botswana or Jordan or South Africa or Brazil or Kazakhstan or St Lucia or Bulgaria or Lebanon or St Vincent) and the Grenadines) or China or Libya or Suriname or Colombia or Macedonia or Thailand or Costa Rica or Malaysia or Tonga or Cuba or Maldives or Turkey or Dominica or Marshall Islands or Turkmenistan or Dominican Republic or Mauritius or Tuvalu or Equatorial Guinea or Mexico or Venezuela or Ecuador or Montenegro or Argentina or Georgia or Kosovo or Moldova or Panama)) OR AB=((Afghanistan or Guinea Bissau or Sierra Leone or Somalia or Burkina Faso or Democratic People's Republic of Korea or South Sudan or Burundi or Liberia or Syrian Arab Republic or Central African Republic or Madagascar or Chad or Malawi or Tanzania or Comoros or Mali or Togo or Democratic Republic of Congo or Mozambique or Uganda or Eritrea or Nepal or Republic of Yemen or Ethiopia or Niger or Zimbabwe or Gambia or Rwanda or Guinea or Senegal or Sudan) OR (((((Angola or Papua New Guinea or Bangladesh or Kenya or Philippines or Bhutan or Kiribati or Sao Tome) and Principe) or Bolivia or Solomon Islands or Cabo Verde or Kyrgyz Republic or Sri Lanka or Cambodia or Laos or Lao PDR or Cameroon or Lesotho or Republic of Congo or Mauritania or Timor-Leste or Cote dIvoire or Tunisia or Djibouti or Ukraine or Egypt or Mongolia or Uzbekistan or El Salvador or Morocco or Vanuatu or Myanmar or Vietnam or Ghana or Nicaragua or West Bank) and Gaza) or Honduras or Nigeria or Zambia or India or Pakistan or Algeria or Benin or Senegal Republic or Tanzania or Micronesia or Comoros or Nepal or Ewastini or Zimbabwe or Palestine or Iran or Indonesia or Haiti or Tajikistan or Belize or Samoa) OR (((((Albania or Fiji or Namibia or Gabon or American Samoa or Grenada or Paraguay or Armenia or Guatemala or Peru or Azerbaijan or Guyana or Romania or Belarus or Russian Federation or Iraq or Bosnia) and Herzegovina) or Jamaica or Serbia or Botswana or Jordan or South Africa or Brazil or Kazakhstan or St Lucia or Bulgaria or Lebanon or St Vincent) and the Grenadines) or China or Libya or Suriname or Colombia or Macedonia or Thailand or Costa Rica or Malaysia or Tonga or Cuba or Maldives or Turkey or Dominica or Marshall Islands or Turkmenistan or Dominican Republic or Mauritius or Tuvalu or Equatorial Guinea or Mexico or Venezuela or Ecuador or Montenegro or Argentina or Georgia or Kosovo or Moldova or Panama)) |
| 12 | TI=(((“Low income countries” or “Middle income countries” or Low) and “middle income countries”) or “Global south” or “Least developed countries” or “Under developed countries” or “Third world countries” or “Second world countries” or “Resource limited countries”) OR AB=(((“Low income countries” or “Middle income countries” or Low) and “middle income countries”) or “Global south” or “Least developed countries” or “Under developed countries” or “Third world countries” or “Second world countries” or “Resource limited countries”) |
| 13 | #3 or #4 or #5 or #6 or #7 or #8 or #9 or #10 |
| 14 | #11 or #12 |
| 15 | TI=(contracepti* or abortion or fertility or antenatal or intrapartum or postnatal or menstru*) OR AB= (contracepti* or abortion or fertility or antenatal or intrapartum or postnatal or menstru*) |
| 16 | #13 or #15 |
| 17 | #1 and #2 and #14 and #16 |

**Database:** Global Medicus Index

| **Search #** | **Terms searched** |
| --- | --- |
| 1 | ((mh:("urban health")) OR (mh:("urban health service")) OR (mh:("urban health services")) OR (mh:("urban areas")) OR (mh:("urban")) OR (mh:("urban hospital")) OR (mh:("urban hospitals")) OR (mh:("urban population")) OR (tw:(urban)) OR (tw:(city)) OR (tw:(cities)) OR (tw:(municipal))) |
| 2 | ((mh:("refugee")) OR (mh:("refugee camp")) OR (mh:("refugee camps")) OR (mh:("refugee political")) OR (mh:("refugee-health")) OR (mh:("refugeehealth")) OR (mh:("refugee/asylum")) OR (mh:("refugee/asylum-seeker")) OR (mh:("refugee/displaced")) OR (mh:("refugeeasylumseeker")) OR (mh:("refugeeasylum")) OR (mh:("refugeedisplaced")) OR (mh:("migrant/refugee")) OR (tw:(refugee)) OR (tw:(migrant)) OR (tw:(displace*)) OR (tw:(asylum))) |
| 5 | ((tw:("developing countries")) OR (tw:("developing nation")) OR (tw:("developing nations")) OR (tw:("third-world countries")) OR (tw:("third-world country")) OR (tw:("third-world nations")) OR (tw:("third world nation" )) OR (tw:("under-developed countries")) OR (tw:("under-developed country")) OR (tw:("under-developed nation")) OR (tw:("under-developed nations")) OR (mh:("developing countries")) OR (mh:("developing nation")) OR (mh:("developing nations")) OR (mh:("third-world countries")) OR (mh:("third-world country")) OR (mh:("third-world nations")) OR (mh:("third world nation" )) OR (mh:("under-developed countries")) OR (mh:("under-developed country")) OR (mh:("under-developed nation")) OR (mh:("under-developed nations"))) |
| 6 | ((tw:("abuse intimate partner")) OR (tw:("chlamydia infection")) OR (tw:("rape" )) OR (tw:("chlamydia")) OR (tw:("chlamydia infections")) OR (tw:("syphilis")) OR (tw:("hiv")) OR (tw:("hiv (human immunodeficiency virus)")) OR (tw:("std")) OR (tw:("gonorrhea")) OR (tw:("venereal disease")) OR (tw:("gender-based violence")) OR (tw:("intimate partner abuse")) OR (tw:("intimate partner violence")) OR (mh:("abuse intimate partner")) OR (mh:("chlamydia infection")) OR (mh:("rape" )) OR (mh:("chlamydia")) OR (mh:("chlamydia infections")) OR (mh:("syphilis")) OR (mh:("hiv")) OR (mh:("hiv (human immunodeficiency virus)")) OR (mh:("std")) OR (mh:("gonorrhea")) OR (mh:("venereal disease")) OR (mh:("gender-based violence")) OR (mh:("intimate partner abuse")) OR (mh:("intimate partner violence"))) |
| 8 | ((mh:("urban health")) OR (mh:("urban health service")) OR (mh:("urban health services")) OR (mh:("urban areas")) OR (mh:("urban")) OR (mh:("urban hospital")) OR (mh:("urban hospitals")) OR (mh:("urban population")) OR (tw:(urban)) OR (tw:(city)) OR (tw:(cities)) OR (tw:(municipal))) AND ((mh:("refugee")) OR (mh:("refugee camp")) OR (mh:("refugee camps")) OR (mh:("refugee political")) OR (mh:("refugee-health")) OR (mh:("refugeehealth")) OR (mh:("refugee/asylum")) OR (mh:("refugee/asylum-seeker")) OR (mh:("refugee/displaced")) OR (mh:("refugeeasylumseeker")) OR (mh:("refugeeasylum")) OR (mh:("refugeedisplaced")) OR (mh:("migrant/refugee")) OR (tw:(refugee)) OR (tw:(migrant)) OR (tw:(displace*)) OR (tw:(asylum))) AND ((tw:("developing countries")) OR (tw:("developing nation")) OR (tw:("developing nations")) OR (tw:("third-world countries")) OR (tw:("third-world country")) OR (tw:("third-world nations")) OR (tw:("third world nation" )) OR (tw:("under-developed countries")) OR (tw:("under-developed country")) OR (tw:("under-developed nation")) OR (tw:("under-developed nations")) OR (mh:("developing countries")) OR (mh:("developing nation")) OR (mh:("developing nations")) OR (mh:("third-world countries")) OR (mh:("third-world country")) OR (mh:("third-world nations")) OR (mh:("third world nation" )) OR (mh:("under-developed countries")) OR (mh:("under-developed country")) OR (mh:("under-developed nation")) OR (mh:("under-developed nations"))) AND ((tw:("abuse intimate partner")) OR (tw:("chlamydia infection")) OR (tw:("rape" )) OR (tw:("chlamydia")) OR (tw:("chlamydia infections")) OR (tw:("syphilis")) OR (tw:("hiv")) OR (tw:("hiv (human immunodeficiency virus)")) OR (tw:("std")) OR (tw:("gonorrhea")) OR (tw:("venereal disease")) OR (tw:("gender-based violence")) OR (tw:("intimate partner abuse")) OR (tw:("intimate partner violence")) OR (mh:("abuse intimate partner")) OR (mh:("chlamydia infection")) OR (mh:("rape" )) OR (mh:("chlamydia")) OR (mh:("chlamydia infections")) OR (mh:("syphilis")) OR (mh:("hiv")) OR (mh:("hiv (human immunodeficiency virus)")) OR (mh:("std")) OR (mh:("gonorrhea")) OR (mh:("venereal disease")) OR (mh:("gender-based violence")) OR (mh:("intimate partner abuse")) OR (mh:("intimate partner violence"))) OR ((tw:("developing countries")) OR (tw:("developing nation")) OR (tw:("developing nations")) OR (tw:("third-world countries")) OR (tw:("third-world country")) OR (tw:("third-world nations")) OR (tw:("third world nation" )) OR (tw:("under-developed countries")) OR (tw:("under-developed country")) OR (tw:("under-developed nation")) OR (tw:("under-developed nations")) OR (mh:("developing countries")) OR (mh:("developing nation")) OR (mh:("developing nations")) OR (mh:("third-world countries")) OR (mh:("third-world country")) OR (mh:("third-world nations")) OR (mh:("third world nation" )) OR (mh:("under-developed countries")) OR (mh:("under-developed country")) OR (mh:("under-developed nation")) OR (mh:("under-developed nations")))  AND  ((mh:("refugee")) OR (mh:("refugee camp")) OR (mh:("refugee camps")) OR (mh:("refugee political")) OR (mh:("refugee-health")) OR (mh:("refugeehealth")) OR (mh:("refugee/asylum")) OR (mh:("refugee/asylum-seeker")) OR (mh:("refugee/displaced")) OR (mh:("refugeeasylumseeker")) OR (mh:("refugeeasylum")) OR (mh:("refugeedisplaced")) OR (mh:("migrant/refugee")) OR (tw:(refugee)) OR (tw:(migrant)) OR (tw:(displace*)) OR (tw:(asylum)))  AND  ((mh:("urban health")) OR (mh:("urban health service")) OR (mh:("urban health services")) OR (mh:("urban areas")) OR (mh:("urban")) OR (mh:("urban hospital")) OR (mh:("urban hospitals")) OR (mh:("urban population")) OR (tw:(urban)) OR (tw:(city)) OR (tw:(cities)) OR (tw:(municipal)))  AND  (mh:(contraceptives or reproductive health or pregnancy or antenatal or postpartum or fertility or menstruation)) OR (tw:(contracepti* or abortion or fertility or antenatal or intrapartum or postnatal or menstru*)) |

**Database:** IBSS

((((MAINSUBJECT.EXACT("Sex crimes") OR MAINSUBJECT.EXACT("Womens health") OR MAINSUBJECT.EXACT("Rape") OR MAINSUBJECT.EXACT("Sexual health") OR MAINSUBJECT.EXACT("Sex education")) OR (MAINSUBJECT.EXACT("Domestic violence") OR MAINSUBJECT.EXACT("Family planning")) OR (MAINSUBJECT.EXACT("Syphilis") OR MAINSUBJECT.EXACT("Gonorrhea") OR MAINSUBJECT.EXACT("Sexually transmitted diseases STD") OR MAINSUBJECT.EXACT("Chlamydia") OR MAINSUBJECT.EXACT("Human immunodeficiency virus HIV"))) OR TIAB(((domestic N3 (abus* OR violen*)) OR ((partner* OR spous* OR wife OR wives) N3 (abuse* OR violen*)) OR "gender-based violence" OR rape OR (sex N3 (abus* OR offen* OR health OR educat* OR counsel*)) OR "family planning" OR (("sexually transmitted" OR venereal) N3 (diseas* OR infect*)) OR (sti OR stis) OR (std or stds) OR (("human immunodeficiency virus" OR HIV) N3 (infect*))) OR syphilis OR chlamydia OR gonorrhea)) AND ((MAINSUBJECT.EXACT("Developing countries LDCs") OR ("Low income countries" OR "Middle income countries") OR ("Low and middle income countries" OR "Least developed countries" OR "Under developed countries" OR "Third world countries" OR "Second world countries" OR "Resource limited countries")) OR TIAB((Afghanistan or Guinea Bissau or Sierra Leone or Somalia or Burkina Faso or Democratic People's Republic of Korea or South Sudan or Burundi or Liberia or Syrian Arab Republic or Central African Republic or Madagascar or Chad or Malawi or Tanzania or Comoros or Mali or Togo or Democratic Republic of Congo or Mozambique or Uganda or Eritrea or Nepal or Republic of Yemen or Ethiopia or Niger or Zimbabwe or Gambia or Rwanda or Guinea or Senegal or Sudan) OR (((((Angola or Papua New Guinea or Bangladesh or Kenya or Philippines or Bhutan or Kiribati or Sao Tome) and Principe) or Bolivia or Solomon Islands or Cabo Verde or Kyrgyz Republic or Sri Lanka or Cambodia or Laos or Lao PDR or Cameroon or Lesotho or Republic of Congo or Mauritania or Timor-Leste or Cote dIvoire or Tunisia or Djibouti or Ukraine or Egypt or Mongolia or Uzbekistan or El Salvador or Morocco or Vanuatu or Myanmar or Vietnam or Ghana or Nicaragua or West Bank) and Gaza) or Honduras or Nigeria or Zambia or India or Pakistan or Algeria or Benin or Senegal Republic or Tanzania or Micronesia or Comoros or Nepal or Ewastini or Zimbabwe or Palestine or Iran or Indonesia or Haiti or Tajikistan or Belize or Samoa) OR (((((Albania or Fiji or Namibia or Gabon or American Samoa or Grenada or Paraguay or Armenia or Guatemala or Peru or Azerbaijan or Guyana or Romania or Belarus or Russian Federation or Iraq or Bosnia) and Herzegovina) or Jamaica or Serbia or Botswana or Jordan or South Africa or Brazil or Kazakhstan or St Lucia or Bulgaria or Lebanon or St Vincent) and the Grenadines) or China or Libya or Suriname or Colombia or Macedonia or Thailand or Costa Rica or Malaysia or Tonga or Cuba or Maldives or Turkey or Dominica or Marshall Islands or Turkmenistan or Dominican Republic or Mauritius or Tuvalu or Equatorial Guinea or Mexico or Venezuela or Ecuador or Montenegro or Argentina or Georgia or Kosovo or Moldova or Panama))) AND (((TIAB(urban OR (cities or city) OR municipal*) OR (MAINSUBJECT.EXACT("Urban areas") OR MAINSUBJECT.EXACT("Urban health care") OR MAINSUBJECT.EXACT("Rural urban migration"))) AND ((Refugee* OR displace* OR asylum OR migrant) OR (MAINSUBJECT.EXACT("Refugee camps") OR MAINSUBJECT.EXACT("Refugees")))) OR (TIAB(urban OR (cities or city) OR municipal*) N3 TIAB(Refugee* OR displace* OR asylum OR migrant)))) OR (((MAINSUBJECT.EXACT("Birth control") OR MAINSUBJECT.EXACT("Reproductive health") OR MAINSUBJECT.EXACT("Postpartum period") OR MAINSUBJECT.EXACT("Pregnancy") OR MAINSUBJECT.EXACT("Prenatal care") OR MAINSUBJECT.EXACT("Menstruation") OR MAINSUBJECT.EXACT("Fertility")) OR (TIAB(contracepti* or abortion or fertility or antenatal or intrapartum or postnatal or menstru*))) AND ((MAINSUBJECT.EXACT("Developing countries LDCs") OR ("Low income countries" OR "Middle income countries") OR ("Low and middle income countries" OR "Least developed countries" OR "Under developed countries" OR "Third world countries" OR "Second world countries" OR "Resource limited countries")) OR TIAB((Afghanistan or Guinea Bissau or Sierra Leone or Somalia or Burkina Faso or Democratic People's Republic of Korea or South Sudan or Burundi or Liberia or Syrian Arab Republic or Central African Republic or Madagascar or Chad or Malawi or Tanzania or Comoros or Mali or Togo or Democratic Republic of Congo or Mozambique or Uganda or Eritrea or Nepal or Republic of Yemen or Ethiopia or Niger or Zimbabwe or Gambia or Rwanda or Guinea or Senegal or Sudan) OR (((((Angola or Papua New Guinea or Bangladesh or Kenya or Philippines or Bhutan or Kiribati or Sao Tome) and Principe) or Bolivia or Solomon Islands or Cabo Verde or Kyrgyz Republic or Sri Lanka or Cambodia or Laos or Lao PDR or Cameroon or Lesotho or Republic of Congo or Mauritania or Timor-Leste or Cote dIvoire or Tunisia or Djibouti or Ukraine or Egypt or Mongolia or Uzbekistan or El Salvador or Morocco or Vanuatu or Myanmar or Vietnam or Ghana or Nicaragua or West Bank) and Gaza) or Honduras or Nigeria or Zambia or India or Pakistan or Algeria or Benin or Senegal Republic or Tanzania or Micronesia or Comoros or Nepal or Ewastini or Zimbabwe or Palestine or Iran or Indonesia or Haiti or Tajikistan or Belize or Samoa) OR (((((Albania or Fiji or Namibia or Gabon or American Samoa or Grenada or Paraguay or Armenia or Guatemala or Peru or Azerbaijan or Guyana or Romania or Belarus or Russian Federation or Iraq or Bosnia) and Herzegovina) or Jamaica or Serbia or Botswana or Jordan or South Africa or Brazil or Kazakhstan or St Lucia or Bulgaria or Lebanon or St Vincent) and the Grenadines) or China or Libya or Suriname or Colombia or Macedonia or Thailand or Costa Rica or Malaysia or Tonga or Cuba or Maldives or Turkey or Dominica or Marshall Islands or Turkmenistan or Dominican Republic or Mauritius or Tuvalu or Equatorial Guinea or Mexico or Venezuela or Ecuador or Montenegro or Argentina or Georgia or Kosovo or Moldova or Panama))) AND (((TIAB(urban OR (cities or city) OR municipal*) OR (MAINSUBJECT.EXACT("Urban areas") OR MAINSUBJECT.EXACT("Urban health care") OR MAINSUBJECT.EXACT("Rural urban migration"))) AND ((Refugee* OR displace* OR asylum OR migrant) OR (MAINSUBJECT.EXACT("Refugee camps") OR MAINSUBJECT.EXACT("Refugees")))) OR (TIAB(urban OR (cities or city) OR municipal*) N3 TIAB(Refugee* OR displace* OR asylum OR migrant))))

**Database:** ASSIA

(((TIAB(urban OR (cities OR city) OR municipal*) N3 TIAB(Refugee* OR displace* OR asylum OR migrant)) OR ((TIAB(urban OR cities OR city OR municipal*) OR (MAINSUBJECT.EXACT("Rural-Urban migrants") OR MAINSUBJECT.EXACT("Rural-Urban migration") OR MAINSUBJECT.EXACT("Urban areas"))) AND (TIAB(Refugee* OR displace* OR asylum OR migrant) OR (MAINSUBJECT.EXACT("Refugee camps") OR MAINSUBJECT.EXACT("Refugees"))))) AND ((MAINSUBJECT.EXACT("Sexual assault") OR MAINSUBJECT.EXACT("Rape") OR MAINSUBJECT.EXACT("Sexual health") OR MAINSUBJECT.EXACT("Sexual health education") OR MAINSUBJECT.EXACT("Domestic violence") OR MAINSUBJECT.EXACT("Family planning") OR MAINSUBJECT.EXACT("Syphilis") OR MAINSUBJECT.EXACT("Gonorrhea") OR MAINSUBJECT.EXACT("Sexually transmitted diseases") OR MAINSUBJECT.EXACT("Chlamydia trachomatis") OR MAINSUBJECT.EXACT("HIV") OR MAINSUBJECT.EXACT("Sexual health promotion") OR MAINSUBJECT.EXACT("Sexual health services")) OR TIAB(((domestic N3 (abus* OR violen*)) OR ((partner* OR spous* OR wife OR wives) N3 (abuse* OR violen*)) OR "gender-based violence" OR rape OR (sex N3 (abus* OR offen* OR health OR educat* OR counsel*)) OR "family planning" OR (("sexuallly transmitted" OR venereal) N3 (diseas* OR infect*)) OR (sti OR stis) OR (std OR stds) OR (("human immunodeficiency virus" OR HIV) N3 (infect*))) OR syphilis OR chlamydia OR gonorrhea)) AND (TIAB((Afghanistan OR Guinea Bissau OR Sierra Leone OR Somalia OR Burkina Faso OR Democratic People's Republic of Korea OR South Sudan OR Burundi OR Liberia OR Syrian Arab Republic OR Central African Republic OR Madagascar OR Chad OR Malawi OR Tanzania OR Comoros OR Mali OR Togo OR Democratic Republic of Congo OR Mozambique OR Uganda OR Eritrea OR Nepal OR Republic of Yemen OR Ethiopia OR Niger OR Zimbabwe OR Gambia OR Rwanda OR Guinea OR Senegal OR Sudan) OR (((((Angola OR Papua New Guinea OR Bangladesh OR Kenya OR Philippines OR Bhutan OR Kiribati OR Sao Tome) AND Principe) OR Bolivia OR Solomon Islands OR Cabo Verde OR Kyrgyz Republic OR Sri Lanka OR Cambodia OR Laos OR Lao PDR OR Cameroon OR Lesotho OR Republic of Congo OR Mauritania OR Timor-Leste OR Cote dIvoire OR Tunisia OR Djibouti OR Ukraine OR Egypt OR Mongolia OR Uzbekistan OR El Salvador OR Morocco OR Vanuatu OR Myanmar OR Vietnam OR Ghana OR Nicaragua OR West Bank) AND Gaza) OR Honduras OR Nigeria OR Zambia OR India OR Pakistan OR Algeria OR Benin OR Senegal Republic OR Tanzania OR Micronesia OR Comoros OR Nepal OR Ewastini OR Zimbabwe OR Palestine OR Iran OR Indonesia OR Haiti OR Tajikistan OR Belize OR Samoa) OR (((((Albania OR Fiji OR Namibia OR Gabon OR American Samoa OR Grenada OR Paraguay OR Armenia OR Guatemala OR Peru OR Azerbaijan OR Guyana OR Romania OR Belarus OR Russian Federation OR Iraq OR Bosnia) AND Herzegovina) OR Jamaica OR Serbia OR Botswana OR Jordan OR South Africa OR Brazil OR Kazakhstan OR St Lucia OR Bulgaria OR Lebanon OR St Vincent) AND the Grenadines) OR China OR Libya OR Suriname OR Colombia OR Macedonia OR Thailand OR Costa Rica OR Malaysia OR Tonga OR Cuba OR Maldives OR Turkey OR Dominica OR Marshall Islands OR Turkmenistan OR Dominican Republic OR Mauritius OR Tuvalu OR Equatorial Guinea OR Mexico OR Venezuela OR Ecuador OR Montenegro OR Argentina OR Georgia OR Kosovo OR Moldova OR Panama)) OR (MAINSUBJECT.EXACT("Least developed countries") OR MAINSUBJECT.EXACT("Developing countries")))) OR (((MAINSUBJECT.EXACT("Contraceptives") OR MAINSUBJECT.EXACT("Antenatal care") OR MAINSUBJECT.EXACT("Contraception") OR MAINSUBJECT.EXACT("Reproductive health") OR MAINSUBJECT.EXACT("Fertility") OR MAINSUBJECT.EXACT("Antenatal") OR MAINSUBJECT.EXACT("Pregnancy") OR MAINSUBJECT.EXACT("Postpartum women") OR MAINSUBJECT.EXACT("Menstruation")) OR TIAB(contracepti* or abortion or fertility or antenatal or intrapartum or postnatal or menstru*))

AND ((TIAB(urban OR (cities OR city) OR municipal*) N3 TIAB(Refugee* OR displace* OR asylum OR migrant)) OR ((TIAB(urban OR cities OR city OR municipal*) OR (MAINSUBJECT.EXACT("Rural-Urban migrants") OR MAINSUBJECT.EXACT("Rural-Urban migration") OR MAINSUBJECT.EXACT("Urban areas"))) AND (TIAB(Refugee* OR displace* OR asylum OR migrant) OR (MAINSUBJECT.EXACT("Refugee camps") OR MAINSUBJECT.EXACT("Refugees"))))) AND (TIAB((Afghanistan OR Guinea Bissau OR Sierra Leone OR Somalia OR Burkina Faso OR Democratic People's Republic of Korea OR South Sudan OR Burundi OR Liberia OR Syrian Arab Republic OR Central African Republic OR Madagascar OR Chad OR Malawi OR Tanzania OR Comoros OR Mali OR Togo OR Democratic Republic of Congo OR Mozambique OR Uganda OR Eritrea OR Nepal OR Republic of Yemen OR Ethiopia OR Niger OR Zimbabwe OR Gambia OR Rwanda OR Guinea OR Senegal OR Sudan) OR (((((Angola OR Papua New Guinea OR Bangladesh OR Kenya OR Philippines OR Bhutan OR Kiribati OR Sao Tome) AND Principe) OR Bolivia OR Solomon Islands OR Cabo Verde OR Kyrgyz Republic OR Sri Lanka OR Cambodia OR Laos OR Lao PDR OR Cameroon OR Lesotho OR Republic of Congo OR Mauritania OR Timor-Leste OR Cote dIvoire OR Tunisia OR Djibouti OR Ukraine OR Egypt OR Mongolia OR Uzbekistan OR El Salvador OR Morocco OR Vanuatu OR Myanmar OR Vietnam OR Ghana OR Nicaragua OR West Bank) AND Gaza) OR Honduras OR Nigeria OR Zambia OR India OR Pakistan OR Algeria OR Benin OR Senegal Republic OR Tanzania OR Micronesia OR Comoros OR Nepal OR Ewastini OR Zimbabwe OR Palestine OR Iran OR Indonesia OR Haiti OR Tajikistan OR Belize OR Samoa) OR (((((Albania OR Fiji OR Namibia OR Gabon OR American Samoa OR Grenada OR Paraguay OR Armenia OR Guatemala OR Peru OR Azerbaijan OR Guyana OR Romania OR Belarus OR Russian Federation OR Iraq OR Bosnia) AND Herzegovina) OR Jamaica OR Serbia OR Botswana OR Jordan OR South Africa OR Brazil OR Kazakhstan OR St Lucia OR Bulgaria OR Lebanon OR St Vincent) AND the Grenadines) OR China OR Libya OR Suriname OR Colombia OR Macedonia OR Thailand OR Costa Rica OR Malaysia OR Tonga OR Cuba OR Maldives OR Turkey OR Dominica OR Marshall Islands OR Turkmenistan OR Dominican Republic OR Mauritius OR Tuvalu OR Equatorial Guinea OR Mexico OR Venezuela OR Ecuador OR Montenegro OR Argentina OR Georgia OR Kosovo OR Moldova OR Panama)) OR (MAINSUBJECT.EXACT("Least developed countries") OR MAINSUBJECT.EXACT("Developing countries"))))
